# Unravelling the Complex Interplay of Transcription Factors Orchestrating Seed Oil Content in *Brassica napus* L.

**DOI:** 10.3390/ijms22031033

**Published:** 2021-01-21

**Authors:** Abirami Rajavel, Selina Klees, Johanna-Sophie Schlüter, Hendrik Bertram, Kun Lu, Armin Otto Schmitt, Mehmet Gültas

**Affiliations:** 1 Breeding Informatics Group, Department of Animal Sciences, Georg-August University, Margarethe von Wrangell-Weg 7, 37075 Göttingen, Germany; abirami.rajavel@uni-goettingen.de (A.R.); selina.klees@uni-goettingen.de (S.K.); j.schlueter01@stud.uni-goettingen.de (J.-S.S.); hendrik.bertram@stud.uni-goettingen.de (H.B.); armin.schmitt@uni-goettingen.de (A.O.S.); 2 College of Agronomy and Biotechnology, Southwest University, Beibei, Chongqing 400715, China; drlukun@swu.edu.cn; 3 Academy of Agricultural Sciences, Southwest University, Beibei, Chongqing 400715, China; 4 State Cultivation Base of Crop Stress Biology, Southern Mountainous Land of Southwest University, Beibei, Chongqing 400715, China; 5 Center for Integrated Breeding Research (CiBreed), Albrecht-Thaer-Weg 3, Georg-August University, 37075 Göttingen, Germany

**Keywords:** *Brassica napus*, MEGs, seed oil content, oil synthesis, transcription factor cooperation

## Abstract

Transcription factors (TFs) and their complex interplay are essential for directing specific genetic programs, such as responses to environmental stresses, tissue development, or cell differentiation by regulating gene expression. Knowledge regarding TF–TF cooperations could be promising in gaining insight into the developmental switches between the cultivars of *Brassica napus* L., namely Zhongshuang11 (ZS11), a double-low accession with high-oil- content, and Zhongyou821 (ZY821), a double-high accession with low-oil-content. In this regard, we analysed a time series RNA-seq data set of seed tissue from both of the cultivars by mainly focusing on the monotonically expressed genes (MEGs). The consideration of the MEGs enables the capturing of multi-stage progression processes that are orchestrated by the cooperative TFs and, thus, facilitates the understanding of the molecular mechanisms determining seed oil content. Our findings show that TF families, such as NAC, MYB, DOF, GATA, and HD-ZIP are highly involved in the seed developmental process. Particularly, their preferential partner choices as well as changes in their gene expression profiles seem to be strongly associated with the differentiation of the oil content between the two cultivars. These findings are essential in enhancing our understanding of the genetic programs in both cultivars and developing novel hypotheses for further experimental studies.

## 1. Introduction

Oil crops have been gaining great economic importance in agriculture as well as in the trade world during the past years [1,2], and the consumption of vegetable oil is anticipated to double by the year 2030 [3]. *Brassica napus* L. (rapeseed or canola) is the third largest source of oilseed crop, which is widely cultivated across the globe [4,5,6]. The seeds of *B. napus* are rich in oil content and fatty acids, which include primarily oleic and linoleic acid [7]. However, erucic acid and glucosinolates are anti-nutritive compounds that are present in the *B. napus* seeds that are not desirable for human consumption or as fodder for animal consumption [8,9]. Therefore, enhancing the seed quality with improved oil content has become the major selective trait for rapeseed breeding programs due to the growing global demand for oil production, for their use as bio-fuel, animal fodder, and vegetable oil [10,11,12].

The seeds of *B. napus* are an excellent reservoir of triacylglycerol (TAG), the primary storage form of oil that is essential for the seedling growth followed by seedling germination [13,14,15,16,17,18]. Recent studies have proposed that the oil content of the seeds could be enhanced by varying the expression levels of individual or a combination of genes encoding transcription factors/enzymes that are associated with TAG metabolism [14,19]. To this end, transcriptome studies have been extensively carried out in order to understand the underlying molecular mechanism regulating the seed oil content of *B. napus* [20,21,22,23]. For this purpose, Xiao et al. [24] identified candidate genes that are involved in regulating the oil content by combining genome-wide association studies and transcriptome analysis in *B. napus*. They performed a comparison study between two extremely high-oil-content lines and one extremely low-oil-content line and identified differentially expressed genes (DEGs) between the lines, contributing to seed oil content. On the other hand, Qu et al. [25] analysed the metabolic profiles of genes that are involved in the flavanoid synthesis in both yellow- and black-seeded rapeseed accessions at the early, middle, and late stages of seed development, and compared the transcriptional differences between them by RNA-sequencing. Moreover, Niu et al. [26] performed pairwise comparisons and also identified DEGs regulating seed size, colour, and oil content in *Brassica rapa*, by taking the seven developmental stages of the seeds into account. In this regard, a recent study conducted by Lu et al. [27] integrated genome-wide association studies and transcriptome analyses, and mainly focused on the identification of DEGs that are related to environmental adaptation, oil content, seed quality, and ecotype improvement for two cultivars of *B. napus*: a double-low accession with high-oil-content and a double-high accession with low-oil-content. Several of the aforementioned studies specifically focused on the Gene Ontology (GO) categories and pathway enrichment anaylses based on the identified DEG sets, while primarily investigating the biological functions of the DEGs regarding seed oil content.

However, today it is well known that transcription factors (TFs) and their complex interplay ccontrol gene expression. Until now, several studies showed that TFs in plants are key regulatory elements controlling the expression of several genes, thereby regulating a variety of essential biological processes, like growth, tissue development, differentiation, metabolism, homeostasis, and adaptation to the environment [28,29,30,31,32,33,34]. Especially in terms of developmental switches and specifying cellular fate in eukaryotes, the orchestration of cell differentiation changes its direction, depending on the specific gene regulatory events that are governed by TFs and their preferential partner choices (for a review see [35]). Thus, the knowledge regarding TFs and their cooperations could provide new insight into the genetic programs regulating various biological processes.

Despite the rich literature on TFs and their cooperations, today there is still a need to unravel the complex interplay of transcription factors orchestrating the seed oil content in *B. napus*. For this purpose, in this study we analysed a time series RNA-seq data set of seed tissue of two *B. napus* cultivars: Zhongshuang11 (ZS11), a double-low accession with high-oil- content and Zhongyou821 (ZY821), a double-high accession with low-oil-content. Unlike the previous studies [24,25,26,27], we investigated the genes with monotonic expression patterns, known as Monotonically Expressed Genes (MEGs), in order to capture the multi-stage progression during seed development. The consideration of the MEGs is promising for capturing the multi-stage progression processes that are directed by the combinatorial interplay of the TFs [36] and, thus, facilitates the understanding of the molecular mechanisms determining the seed oil content. We computationally identified the interplay between the TFs for both cultivars in order to decipher the gene regulatory mechanisms controlling the specific expression pattern of MEGs. Our results show that a vast majority of the TFs are overlapping in both cultivars, while few TFs changes their partners, which could be controlling the switches of developmental programs regarding the oil content of both cultivars.

## 2. Results and Discussion

### 2.1. Identification and Analysis of MEGs

Analysing the time series data set of *B. napus* seeds from the two cultivars ZS11 and ZY821, we have obtained the MEGs for each cultivar, which are monotonically expressed either in ascending or descending patterns during the seed development. Table 1 provides the numbers of MEGs obtained for each cultivar and the obtained MEGs are listed in Supplementary Table S1. Table 1 shows that there is a comparatively large number of MEGs (both ascending and descending MEGs) for the ZS11 cultivar in comparison to these of the ZY821 cultivar.

**Figure 1:**
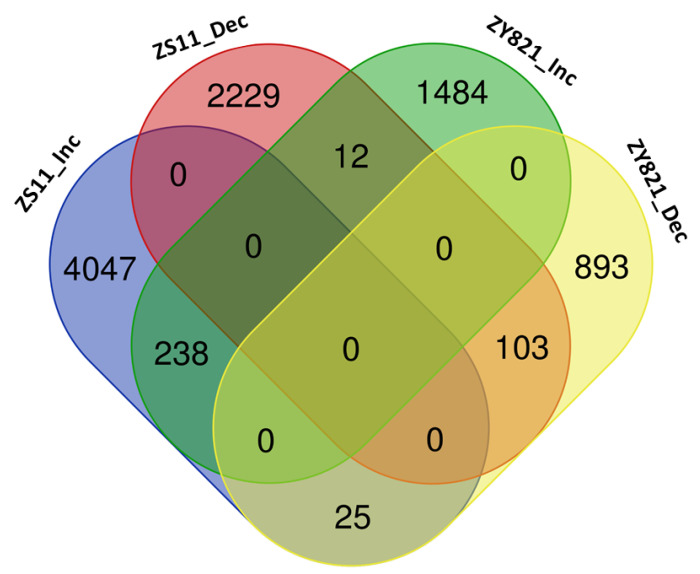
Venn-diagram of the MEGs expressed in ascending and descending orders for the seeds of two cultivars ZS11 and ZY821. (ZS11_Inc: Ascendings MEGs for ZS11; ZS11_Dec: Descending MEGs for ZS11; ZY821_Inc: Ascendings MEGs for ZY821; ZY821_Dec: Descending MEGs for ZY821) (visualised with http://bioinformatics.psb.ugent.be/webtools/Venn/).

Further analysis of the MEGs reveals that, while the vast majority of MEGs are primarily unique to a particular cultivar, only a small number of genes are overlapping between the gene sets of ZS11 and ZY821 (see Figure 1).

Moreover, we performed a gene set enrichment analysis [37] while using the MEGs to obtain deeper insight into their crucial biological functions and clustered these functions based on the GO terms.

The GO enrichment results regarding the MEGs of ZS11 cultivar revealed that the ascending MEGs are significantly enriched mainly in the term “fatty acid metabolism” (see Figure S1), which is highly associated with oil content. Other enriched GO terms, including “sucrose biosynthetic process”, “glycerophospholipid synthetic process”, “sphingolipid metabolic process”, and “galactose metabolic process” are greatly relevant to fatty acid synthesis and accumulation processes. In contrast, for the descending MEGs of the ZS11 cultivar (see Figure S2), the GO term “protein phosphorylation” is significantly enriched that represents particularly the inverse correlation of oil and protein levels [38] in rapeseed. The GO terms indicate that the increasing pattern of MEGs in fatty acid metabolism could contribute to seed oil content of the rapeseed in ZS11 cultivar.

On the other hand, the enrichment analysis of ascending MEGs that were obtained for ZY821 cultivar showed that several significantly enriched GO terms are linked to the “thiamine metabolism”, which is associated with the adaptation to biotic and abiotic stress [39] (see Figure S3). Reversely, for the descending MEGs, GO terms that are related to biological processes, such as “lipid metabolism”, “carbohydrate metabolism”, “GDP-mannose metabolism”, and “sulphur compound metabolism” show that genes that are involved in multiple metabolic processes are following the decreasing pattern in the seeds of ZY821 cultivar during the germination process, especially of lipids (see Figure S4). These results imply that the seeds of ZY821 cultivar might be involved in stress responses while diminishing the other metabolic processes, especially those that are related to oil synthesis.

### 2.2. Cooperative TFs Regulating Seed Developmental Processes of ZS11 and ZY821

**Figure 2:**
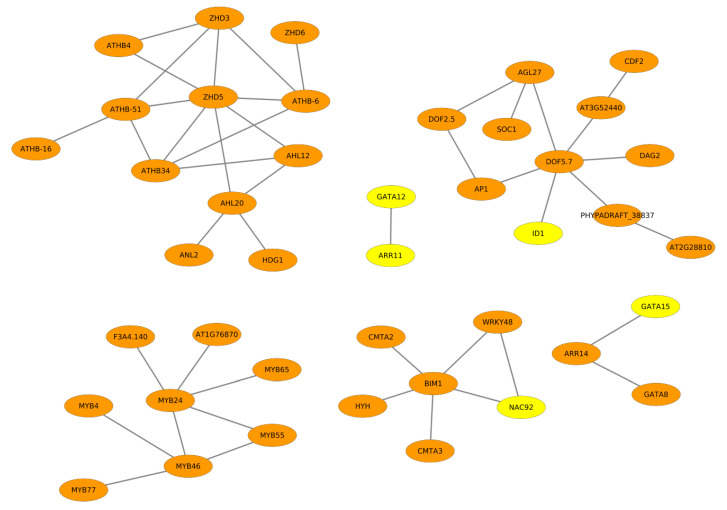
Cooperation network of TF pairs identified for the double low accession ZS11 cultivar. The orange shaded nodes represent the overlapping TFs between ZS11 and ZY821, whereas the yellow shaded nodes represent the TFs that are unique for the ZS11 cultivar.

**Figure 3:**
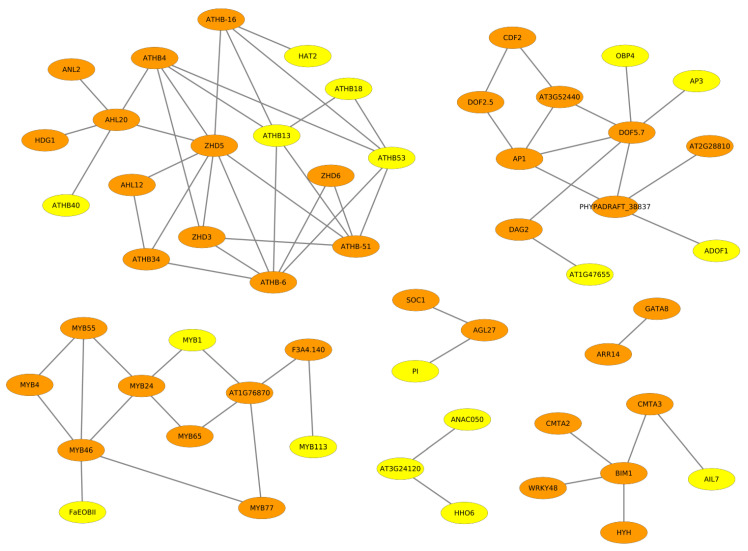
Cooperation network of TF pairs identified for the double high accession ZY821 cultivar. The orange shaded nodes represent the overlapping TFs between ZS11 and ZY821, whereas the yellow shaded nodes represent the TFs that are unique for the ZY821 cultivar.

For the better understanding of the transcriptional regulation underlying oil synthesis in the seeds of *B. napus* and, thereby deciphering specific regulatory programs differentiating between double low and double high cultivars of *B. napus*, we analysed the promoter regions of the MEGs of both cultivars of *B. napus*. Consequently, we identified cooperative transcription factors (TFs) using the algorithm of PC-TraFF, similar to our other studies [40,41,42,43]. The obtained cooperative TF pairs are depicted as cooperation networks in which the elliptical nodes represent the individual TFs, whereas the edges (grey lines in the cooperation networks) represent the cooperation between the TFs (see Figure 2 and Figure 3). The cooperation networks comprise 42 and 54 cooperative TF pairs for ZS11 and ZY821, respectively. The overlapping TFs between the two cooperation networks are shaded in orange and TFs of the cooperation network unique to a particular cultivar are shaded in yellow. Remarkably, a brief analysis of the cooperation networks reveals that there are only five and 17 TFs unique to ZS11 and ZY821, respectively.

### 2.3. Cooperation Network of ZS11 and ZY821

Taking a closer look into the cooperation networks of ZS11 and ZY821 reveals that the majority of the single TFs are overlapping in the networks of both cultivars, while a few of them change their partners. Among several transcription factors in the cooperation networks of ZS11 and ZY821, a majority of them belong predominantly to five TF families: NAC, MYB, DOF, GATA, and the HD-ZIP family. Hence, in our further analysis, we mainly focused on the members of these TF families and their preferential partner choices in order to explain, in detail, their relevance for fatty acid synthesis, transport, and accumulation in the seed tissue of *B. napus* in the two cultivars. The expression profiles of transcription factors that are present in the cooperation networks are provided in Supplementary Information Figures S1–S36.

#### 2.3.1. NAC Family of Transcription Factors

The NAC family members NAC92 and ANAC050 are found with different partners in the cooperation networks of ZS11 and ZY821 (see Figure 2 and Figure 3). This family encodes NAC (NAM, ATAF1, -2, and CUC2) domain transcription factors that are exclusively found in plants and well-studied for their functioning in abiotic stress responses [44,45,46,47,48] and defense mechanisms [44,45,48]. In Figure 2, we observed that, while NAC92 cooperates with the transcription factors BIM1 and WRKY48, the factor ANAC050 cooperates with AT3G24120. Particularly, NAC92 has been reported in several plant species in controlling the age-dependent senescence and seed germination processes [49]. Therefore, it could play a crucial part in determining the seed oil content, as senescence directly affects the quality of seeds. On the other hand, ANAC050 has been studied in transcriptional repression and flowering time control [50] and, thus, its cooperation with AT3G24120 (see Figure 3) could potentially play negative roles in fatty acid accumulation.

**Figure 4:**
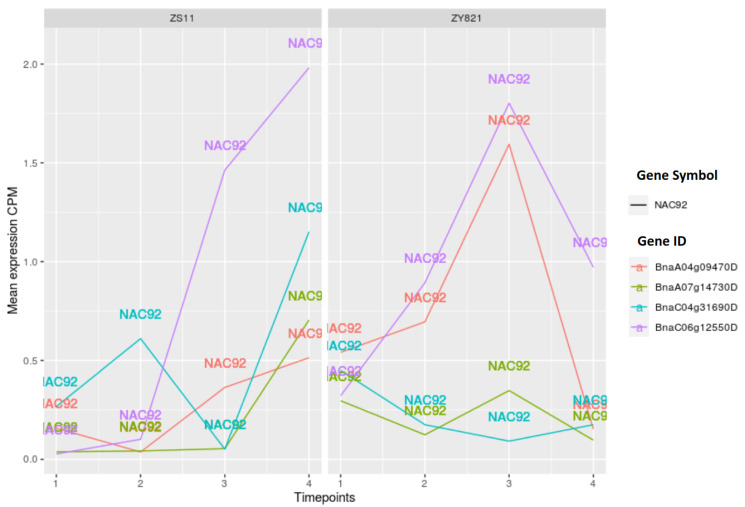
Expression values of NAC92 orthologous genes. Time points 1 to 4 represents day 7, day 10, day 15, and day 45 after flowering.

Moreover, when considering the orthologous genes of NAC92 in *B. napus*, we identified four gene IDs (*BnaA04g09470D*, *BnaA07g14730D*, *BnaC04g31690D*, and *BnaC06g12550D*) while using the Ensembl plant database [51,52]. The gene expression values of these genes show different patterns in both cultivars, as shown in Figure 4. Especially, *BnaC06g12550D* is clearly showing an increasing trend after 10 days of flowering in ZS11 (time point 2 in Figure 4), while its expression level, together with that of *BnaA04g09470D*, is decreasing in ZY821 during the late stage (day 45 after flowering) of seed development. However, the analysis of ANAC050 orthologous genes in *B. napus* reveals that their expression patterns are similar in both of the cultivars (see Supplemetary Information Figure S1).

#### 2.3.2. GATA Transcription Factors

Another interesting family of transcription factors observed in the networks of ZS11 and ZY821 is the GATA family of transcription factors (namely GATA8, GATA12, and GATA15), which contain type-IV zinc finger motifs. GATA transcription factors have been identified in the regulatory regions of the light-responsive genes [53], especially genes that are associated with photosynthesis e.g., the chlorophyll a/b binding protein [54,55]. They play a pivotal role as regulators that are involved in the nitrogen assimilation process in plants [56,57]. Additionally, few members of the GATA family have also been identified as a differentially expressed TF family while comparing high- and low-yielding oil palm [58], which could also explain their major role in rapeseed. In particular, we identified GATA8 with its cooperation partner ARR14 for both cultivars, while GATA12 as well as GATA15 with their cooperation partners ARR11 and ARR14, have only been found for the ZS11 cultivar (see Figure 2 and Figure 3). This finding suggests that, during the seed germination processs, these three GATA family members form dimers with ARR family TFs, which play essential roles in stress responses (involving triacylglycerol) [59]. These TF cooperations in the seed tissue of ZS11 might be directing the regulatory processes that are involved in fatty acid synthesis and accumulation processes.

**Figure 5:**
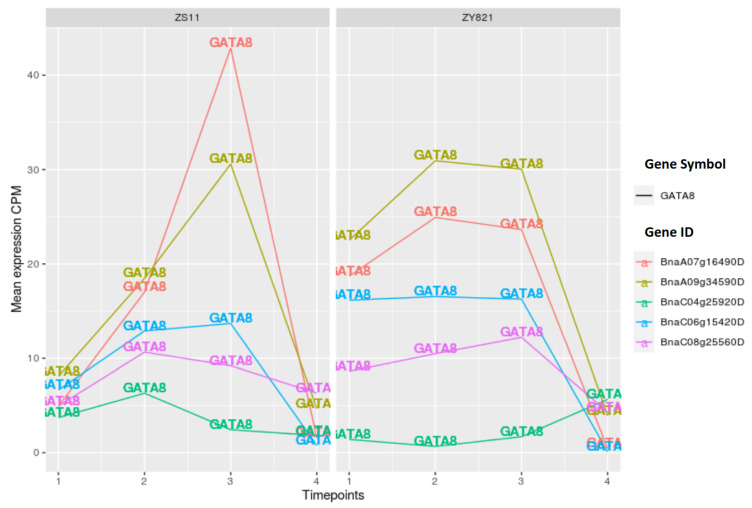
Expression values of GATA8 orthologous genes. Time points 1 to 4 represents day 7, day 10, day 15, and day 45 after flowering.

Furthermore, we observed a strong increment in the expression levels of *BnaA07g16490D* and *BnaA09g34590D* until day 15 after flowering in the ZS11 cultivar in comparison to ZY821 while taking the expression profiles of five GATA8 orthologous genes (*BnaA07g16490D*, *BnaC08g25560D*, *BnaC04g25920D*, *BnaC06g15420D*, and *BnaA09g34590D*) into account (see Figure 5). Interestingly, in both cultivars, the gene expression values of all five orthologous genes abruptly decreases during the late stage of seed development. Similar patterns of the changes in the expression values during the seed development have been obtained for GATA12 and GATA15 orthologous genes (see Supplementary Information Figures S2 and S3).

#### 2.3.3. DOF Family of Transcription Factors

Importantly, we identified several DOF family members (DOF2.5, DOF5.7, DAG2, CDF2, AT2G28810, AT3G52440, OBP4, ADOF1, AT1G47655) in the cooperation networks of ZS11 and ZY821 (see Figure 2 and Figure 3). This family of transcription factors, encoding zinc finger protein, is specific for plants, and it is not found in other eukaryotes [60]. They have been found to be particularly implicated in controlling seed germination, seed storage, and the mobilisation of proteins and fatty acids [61]. Interestingly, DOF2.5/DAG2 acts as a positive regulator of seed germination [62]. Although the functions of DOFs are not well-characterised with regard to seed oil content in *Arabidopsis*, the soybean genes *GmDof4* and *GmDof11* are shown to increase the seed oil content by directly inducing the acetyl CoA carboxylase and long-chain-acyl CoA synthetase synthesis genes [63] that are involved in fatty acid synthesis and metabolism [63,64]. Likewise, the overexpression of *GhDOF1* (*Gossypium hirsutum*) leads to increased lipid levels of cotton [65].

In the cooperation network for ZS11 (see Figure 2), there is unique cooperation between the TFs DOF5.7 and ID1. On the other hand, the factor DOF5.7 with its cooperation partners OBP4 and AP3 have been found to be unique for the network for ZY821 (see Figure 3). Furthermore, we found that the factor DAG2 cooperates with AT1G47655 and ADOF1 (see Figure 3). Given the importance of the DOF family of TFs in influencing the oil content, these preferential partner choices of DOF5.7 could be playing important roles in differentiating the regulatory processes in the seeds of the ZS11 and ZY821 cultivars.

**Figure 6:**
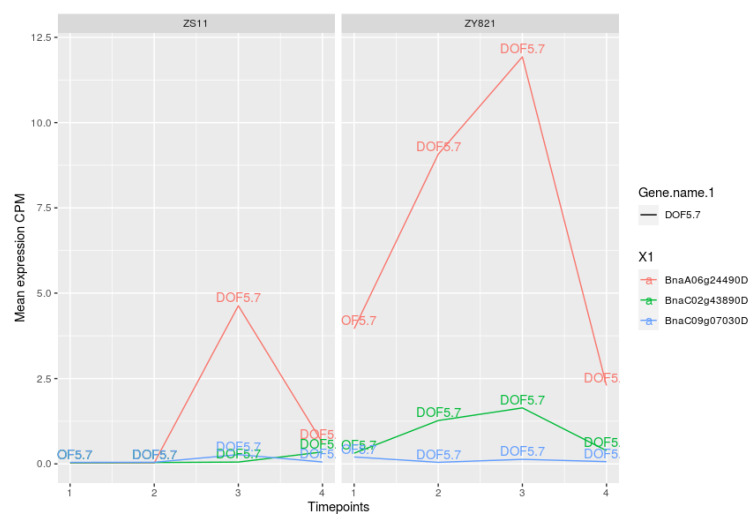
Expression values of DOF5.7 orthologous genes. Time points 1 to 4 represents day 7, day 10, day 15, and day 45 after flowering.

Further, regarding the expression profiles of DOF5.4 orthologous genes (*BnaA06g24490D*, *BnaA02g43890D*, *BnaA09g07030D*), although *BnaA06g24490D* is absent for ZS11 in early stages (<day 10)), its gene expression value is strongly increasing until day 15 after flowering and decreasing after day 15 in both of the cultivars (see Figure 6). The expression profiles of other DOF family members are given in the Supplementary Information Figures S4–S8.

#### 2.3.4. HD-ZIP Family of Transcription Factors

Multiple transcription factors from the cooperation networks, including HDG1, HDG11, ANL2, ATHB-6, ATHB-13, ATHB-40, and ATHB-53, belong to the homeodomain zipper family (see Figure 2 and Figure 3). From this family of proteins, GL2 was the first identified protein that is responsible for the outgrowth of trichome in the epidermis [66] and for contributing to seed oil content in *Arabidopsis* [67]. Additionally, other members of this family have similar functions that are associated with the epidermis [68]. Moreover, the factor ANL2 is implicated in the regulation of anthocyanin accumulation in the shoot and also in the development of root [69]. Several studies have reported the functioning of the HD-ZIP family of transcription factors in the cuticle development. HDG11 has been implicated as a positive regulator of drought stress tolerance [70]. Furthermore, the overexpression of *OCL1* in maize belonging to HD-ZIP family highly influenced the expression levels of various genes that are associated with lipid metabolism [71]. Regarding the functioning of ATHB-6, it has been reported as a regulator of ABA hormone responses and it is also regarded as a target of protein phosphatase ABI1, which is a negative regulator of TAG accumulation and ABA signalling [72,73,74]. Moreover, ATHB-53 is regarded as an auxin-inducible protein and it plays a prominent role in the auxin/cytokinin pathway during root development [75]. However, several members of this family are related to the epidermis development [68], which is also an integral part of the seed coat.

**Figure 7:**
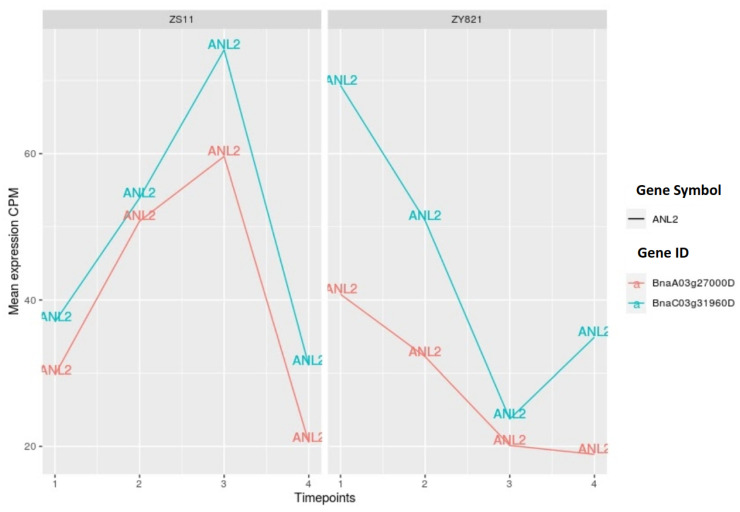
Expression values of ANL2 orthologous genes. Time points 1 to 4 represents day 7, day 10, day 15, and day 45 after flowering.

**Figure 8:**
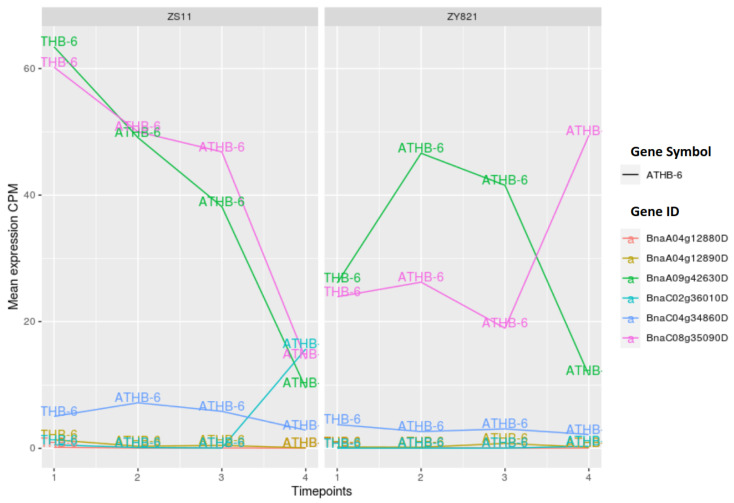
Expression values of ATHB-6 orthologous genes. Time points 1 to 4 represents day 7, day 10, day 15, and day 45 after flowering.

We identified four unique HD-ZIP family members (ATHB-13, ATHB-18, ATHB-40 and ATHB-53) for the ZY821 cultivar while comparing the cooperation networks of the ZS11 and ZY821 cultivars (see Figure 2 and Figure 3). Interestingly, our comparitive analysis reveals that the transcription factors ANL2 and ATHB-6 cooperate with the same partners in both networks. Figure 7 and Figure 8 show the changes in the expression levels of the orthologous genes of both TFs. When considering the expression profiles of ANL2 orthologous genes (*BnaA03g27000D*, *BnaC03g31960D*), the gene expression values of both genes show higher expression levels until day 15 after flowering and they are decreasing after day 15 in ZS11. Whereas, the ANL2 orthologous gene is expressed at high expression levels in an early stage and is declined afterwards during subsequent stages (see Figure 7). In contrary to ANL2, the ATHB-6 orthologous genes (*BnaA09g42630D*, and *BnaC08g35090D*) show higher expression levels in ZS11 in the early stage (day 7) and lower expression level in ZY821. Intriguingly, these expression levels are continuously decreasing in ZS11 during the seed developmental stages. On the other hand, *BnaA09g42630D* and *BnaC08g35090D* are antagonistically expressed to each other in the ZY821 cultivar.

#### 2.3.5. MYB Family of Transcription Factors

In both cooperation networks, we identified the transcription factors MYB1, MYB4, MYB24, MYB46, MYB55, MYB65, MYB77, MYB113, and FaEOBII, which belong to the MYB superfamily. A large number of MYB TFs play central roles in a variety of physiological processes, especially growth, development, synthesis of secondary metabolites, metabolism, and responses to biotic and abiotic stresses [76,77,78,79,80].

Interestingly, the factor MYB24 cooperates with MYB46, MYB55, and MYB65 during seed development in both of the cultivars. Taking its unique cooperations into account, MYB24 forms dimers with F3A4.140 and AT1G76870 in the ZS11 cultivar (see Figure 2). On the other hand, MYB24 is interacting with MYB1 in ZY821 (see Figure 3). Regarding the function of MYB1, it has been characterised as a pivotal positive regulator of the anthocyanin synthesis in onion, thus representing an important player in the flavanoid synthesis pathway on the transcriptional level [81].

A similar role has been studied for MYB113 in purple cauliflower [82] and for FaEOBII in the phenypropanoid pathway in strawberries [83]. Likewise, MYB4 has been reported in *Arabidopsis* for its dual role in the flavanoid biosynthetic pathway, describing the precise regulation of anthocyanin and phenylalanine synthesis [84]. The role of flavonoid snythesis pathway genes in contributing to seed colour that differentiates between high-oil content and low-oil content accessions has been well-reported in [26]. More importantly, the transcription factor MYB46 functions as a master regulator in the secondary wall synthesis, regulating the genes that are involved in the synthesis of three major components (cellulose, hemi cellulose, and lignin) of secondary cell walls [85]. Therefore, it is a modulator in the regulation of defense responses to the fungus *Botrytis cinerea* [86].

**Figure 9:**
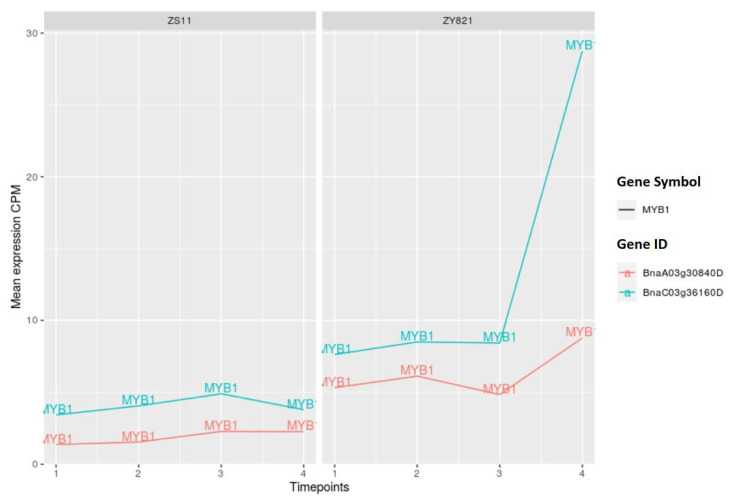
Expression values of MYB1 orthologous genes. Time points 1 to 4 represents day 7, day 10, day 15, and day 45 after flowering.

**Figure 10:**
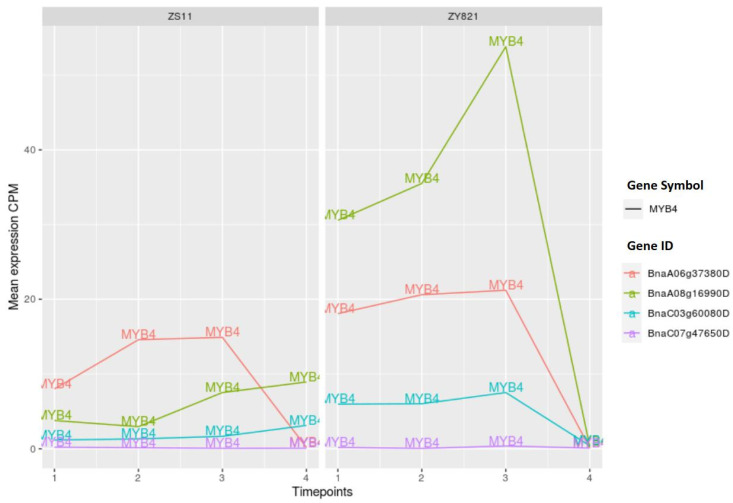
Expression values of MYB4 orthologous genes. Time points 1 to 4 represents day 7, day 10, day 15, and day 45 after flowering.

The gene expression analysis of MYB1 and MYB4 orthologous genes show that the corresponding expression levels are clearly different between ZS11 and ZY821 cultivars. In particular, there is a remarkable increase in the expression level of the MYB1 orthologous genes *BnaC03g63160D* during the late stage after flowering in the ZY821 cultivar (>day 15) (see Figure 9). Another interesting pattern has been observed for the MYB4 orthologous gene *BnaA08g16990D*: While its expression level increases until day 15 after flowering in ZY821 cultivar, it sharply decreases at the late stage (day 45) (see Figure 10).

#### 2.3.6. Other Transcription Factors

There are other crucial transcription factors, like CAMTA2 and CAMTA3, found for both cultivars or ARR11 as well as ID1 found only for the ZS11 cultivar. The roles of these TFs are well studied in biotic and abiotic stress responses [59,87,88,89]. For example, CAMTA2/3 are calmodulin binding transcription factors linking calcium signalling to the induction of defense response genes during abiotic and biotic stress conditions [87,88,89]. They are involved in low temperature and freezing tolerance in *Arabidopsis* [87]. Further, the factor ARR11, encoding the *Arabidopsis* response regulator 11, is implicated for its essential role in mediating abscisic acid and cytokinin signalling pathways and tolerance to drought, thereby it is involved in the generation of drought stress responses [59]. Figure S5 in Supplementary Information gives the expression profile of ARR11. In contrary, the ID1 (Indeterminate Domain) transcription factor found in the cooperation network of ZS11 (see Figure 2) encodes a zinc finger, which has been reported in the regulation of seed development in maize [90,91]. In *Arabidopsis*, it is regarded to activate or inhibit seed germination, with respect to gibberellic acid [90]. Because there is a close association between the stress responses and the contribution of triacylglycerol, which is a major lipid reserve [92], this could explain the contribution of TFs to multiple processes, including fatty acid accumulation, seed germination, and the generation of stress responses.

## 3. Materials and Methods

**Figure 11:**
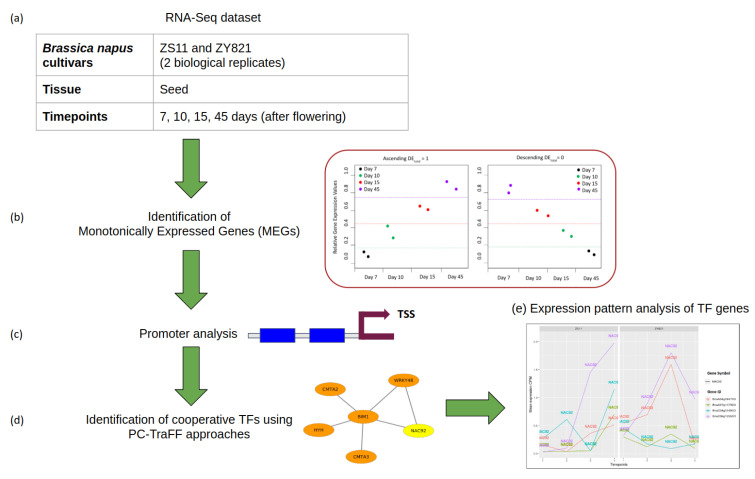
Flowchart of analysis. (**a**) Processing of the RNA-Seq dataset; (**b**) identification of Monotonically Expressed Genes using the MFSelector approach [93]; (**c**) promoter analysis (TSS: transcription start site); (**d**) identification of cooperative transcription factors (TFs) using the PC-TraFF approach [42]; and, (**e**) Expression pattern analysis of TF genes.

Our analysis follows the structure, as presented in Figure 11.

### 3.1. *B. napus* Data Set and Data Preparation

In this study, we use a publicly available time series transcriptomic data set of the seeds that were obtained from two *B. napus* cultivars with different seed oil content, namely Zhongshuang11 (ZS11), a double-low accession with high-oil-content, and Zhongyou821 (ZY821), a double-high accession with low-oil-content. The RNA-sequencing of the seeds from both cultivars at four different time points, namely day 7, day 10, day 15, and day 45 after flowering, with two biological replicates each were generated by Lu et al. [27]. The raw sequencing data are available from the BIG Data Center under the BioProject accession code PRJCA001246. Readers who are interested in learning more about this data set are kindly referred to the original study [27].

Following the original study [27], we mapped the filtered reads to the *B. napus* reference genome version 4.1 (obtained from [94] and available under https://wwwdev.genoscope.cns.fr/brassicanapus/data/) while using STAR 2.4.4a [95]. We then obtained the gene counts from the aligned sequence reads by applying the htseq-count program [96]. In total, the data set comprises raw count values for 80,927 genes and 16 samples (four time points with two biological replicates each for two cultivars). Finally, the raw counts were normalized while using the R function voom with normalization method “cyclicloess” of the package limma (version 3.40.6) [97] in order to obtain the counts-per-million (CPM) normalized values.

### 3.2. Identification of Monotonically Expressed Genes

By applying the MFSelector (monotonic feature selector) approach [93] to the time series data set obtained from RNA-seq consisting of four time points, we identified Monotonically Expressed Genes (MEGs), whose expression patterns are closely linked to the development of the seeds over time in both cultivars. The underlying algorithm of MFSelector compares the expression values of each gene observed for all of the samples between time points in order to assess whether these values follow a strong monotonic pattern. In addition, a permutation test is performed to determine the significance level of these patterns. Consequently, it provides two sets of MEGs with corresponding *p*-values: while the first set contains the genes with significantly monotonically increasing patterns, the second set only includes genes with significantly monotonically decreasing expression values.

In MFSelector, the parameters *permut*, *svdetimes*, and *svdenoise* have to be specified in order to define the level of stringency for monotonicity. Following our previous study [36], we chose *permut* = 100, *svdetimes* = 100, and *svdenoise* = 0.1. Finally, we considered, in our further analysis, only the genes as significant MEGs that have a FDR ≤0.1 and sample variance for discriminating error value ≤1.

### 3.3. Gene Ontology Enrichment Analysis

While using PlantGSEA (http://systemsbiology.cau.edu.cn/PlantGSEA/) [37], we performed the gene set enrichment analysis in order to obtain GO (Gene Ontology) terms on “biological process” regarding the ascending and descending set of MEGs for both ZS11 and ZY821 cultivars. Fisher’s exact test was applied and a GO term was considered to be statistically significant if its corresponding FDR value ≤0.05. The enriched GO terms were visualised as tree map while using REVIGO [98].

### 3.4. Identification of Transcription Factor Cooperations

For the identification of transcription factors (TFs) that cooperatively bind to promoter regions of genes, we applied the PC-TraFF approach [42]. The PC-TraFF is an information theory based methodology that applies the pointwise mutual information (PMI) metric in order to quantify the cooperation level of TFs, according to the co-occurrence of their binding sites in the promoters of the MEGs [42]. Its underlying algorithm consists of six phases and it requires the following input parameters:

- Promoter sequences: Similar to our study [28], we extracted, for each MEG, its corresponding promoter sequence ranging from −500 bp to +100 bp regions relative to a transcription start site while using the reference genome version 4.1 and gene annotation given in [94].
- Transcription Factor Binding Site (TFBS) detection: For the detection of putative TFBSs in the promoter sequences, we employed the MATCH™ program [99] with a non-redundant plant position weight matrix (PWM) library from the JASPAR database [100].
- Pre-defined distances: For the identification of the regular cooperative binding pattern of TFs, the underlying PC-TraFF algorithm requires the pre-defined minimum and maximum distance thresholds between TFBSs. In our analysis, we used the recommended values of a distance ≤20 for the maximum and ≥5 for the minimum distance.

Consequently, the algorithm of the PC-TraFF approach assigns each TF-pair ($T_{a}$ and $T_{b}$) a PMI $\left(T_{a};\;T_{b}\right)$-score and it transforms the PMI $\left(T_{a};\;T_{b}\right)$-score into the *z-score* as a final step. A cooperation between any $T_{a}$ and $T_{b}$ is considered to be significant if they have a *z-score*≥3.

### 3.5. Expression Pattern Analysis of TF Genes

Following the analysis strategy of Zeidler et al. [43], we analysed the changes in the expression values of TF genes during the seed developmental stages to gather knowledge on the combinatorial gene regulation underlying the differentiation of the oil content between the two cultivars. For this purpose, we determined, for each TF, whose gene symbol is often defined in *Arabidopsis thaliana* or *Zea mays*, its orthologous genes in *B. napus* while using the Ensembl plant database [51,52].

## 4. Conclusions

Transcriptional regulation in plants plays a pivotal role in governing a variety of physiological processes. In oil crops, like *B. napus*, a deeper knowledge regarding TFs and their combinatorial interplay sheds light into the regulatory mechanisms that underlie seed oil content, particularly in the accumulation of fatty acids. In our study, by analysing a RNA-seq data set of seed tissue from two *B.napus* accessions, a double-low accession with high-oil-content and a double-high accession with low-oil-content, we identified several TFs and their preferential partner choices, which are likely to influence the quality of seed oil content. Interestingly, some of the TFs have the same cooperation partners in both cultivars, whereas the gene expression patterns of their orthologous genes clearly show distinguishing patterns between the cultivars during the seed development process. To the best of our knowledge, this is the first study performing a systematic analysis to decipher the complex interplay of the TFs that are linked with developmental switches resulting in a higher oil content. Our findings could be promising for deepening the existing knowledge on the transcriptional regulation governing seed oil content notwithstanding the absence of experimental validation. Therefore, further progress from the molecular plant biology end is needed, not only to validate the functions of these TFs, but also for a future perspective on generating novel hypotheses in genetic programs that involve seed oil improvement.

## Figures and Tables

**Table 1:** Numbers of significant Monotonically Expressed Genes in ascending and descending order for the seeds of two cultivars of *B. napus* namely ZS11 and ZY821.

	ZS11		ZY821	
	**Ascending**	**Descending**	**Ascending**	**Descending**
Genes	4310	2344	1734	1021

## Supplementary Materials

The following Figures are available online at https://www.mdpi.com/1422-0067/22/3/1033/s1.

## References

[1] Gunstone F.D., Harwood J.L., Dijkstra A.J. (2007). The Lipid Handbook with CD-ROM.

[2] USDA Oilseeds: World Markets and Trade. https://www.fas.usda.gov/data/oilseeds-world-markets-and-trade.

[3] USDA (2011). Unites States Department of Agriculture Foreign Agricultural Service, Global Agricultural Trade System (GATS), Forest Products Trade Statistics. http://www.fas.usda.gov/gats/default.aspx.

[4] Woodfield H., Harwood J. (2017). Oilseed Crops: Linseed, Rapeseed, Soybean, and Sunflower. Encyclopedia of Applied Plant Sciences.

[5] Gracka A., Jeleń H.H., Majcher M., Siger A., Kaczmarek A. (2016). Flavoromics approach in monitoring changes in volatile compounds of virgin rapeseed oil caused by seed roasting. J. Chromatogr. A.

[6] Liu S., Fan C., Li J., Cai G., Yang Q., Wu J., Yi X., Zhang C., Zhou Y. (2016). A genome-wide association study reveals novel elite allelic variations in seed oil content of *Brassica napus*. Theor. Appl. Genet..

[7] Velasco L., Möllers C., Becker H.C. (1999). Estimation of seed weight, oil content and fatty acid composition in intact single seeds of rapeseed (*Brassica napus* L.) by near-infrared reflectance spectroscopy. Euphytica.

[8] Wittkop B., Snowdon R., Friedt W. (2009). Status and perspectives of breeding for enhanced yield and quality of oilseed crops for Europe. Euphytica.

[9] Snowdon R., Lühs W., Friedt W. (2007). Oilseed rape. Oilseeds.

[10] Abbadi A., Leckband G. (2011). Rapeseed breeding for oil content, quality, and sustainability. Eur. J. Lipid Sci. Technol..

[11] Hannoufa A., Pillai B.V., Chellamma S. (2014). Genetic enhancement of *Brassica napus* seed quality. Transgenic Res..

[12] Harper A.L., Trick M., Higgins J., Fraser F., Clissold L., Wells R., Hattori C., Werner P., Bancroft I. (2012). Associative transcriptomics of traits in the polyploid crop species *Brassica napus*. Nat. Biotechnol..

[13] Knauer S., Holt A.L., Rubio-Somoza I., Tucker E.J., Hinze A., Pisch M., Javelle M., Timmermans M.C., Tucker M.R., Laux T. (2013). A protodermal miR394 signal defines a region of stem cell competence in the Arabidopsis shoot meristem. Dev. Cell.

[14] Tan H., Yang X., Zhang F., Zheng X., Qu C., Mu J., Fu F., Li J., Guan R., Zhang H. (2011). Enhanced seed oil production in canola by conditional expression of *Brassica napus* LEAFY COTYLEDON1 and LEC1-LIKE in developing seeds. Plant Physiol..

[15] Nesi N., Delourme R., Brégeon M., Falentin C., Renard M. (2008). Genetic and molecular approaches to improve nutritional value of *Brassica napus* L. seed. Comptes Rendus Biol..

[16] Murphy D.J., Cummins I., Kang A.S. (1989). Synthesis of the major oil-body membrane protein in developing rapeseed (Brassica napus) embryos. Integration with storage-lipid and storage-protein synthesis and implications for the mechanism of oil-body formation. Biochem. J..

[17] Goldberg R.B., De Paiva G., Yadegari R. (1994). Plant embryogenesis: Zygote to seed. Science.

[18] Huang A.H. (1992). Oil bodies and oleosins in seeds. Annu. Rev. Plant Biol..

[19] Van Erp H., Kelly A.A., Menard G., Eastmond P.J. (2014). Multigene engineering of triacylglycerol metabolism boosts seed oil content in Arabidopsis. Plant Physiol..

[20] Zhu L., Zhao X., Xu Y., Wang Q., Wang H., Wu D., Jiang L. (2020). Effect of germination potential on storage lipids and transcriptome changes in premature developing seeds of oilseed rape (*Brassica napus* L.). Theor. Appl. Genet..

[21] Shahid M., Cai G., Zu F., Zhao Q., Qasim M.U., Hong Y., Fan C., Zhou Y. (2019). Comparative Transcriptome Analysis of Developing Seeds and Silique Wall Reveals Dynamic Transcription Networks for Effective Oil Production in *Brassica napus* L.. Int. J. Mol. Sci..

[22] Jiang J., Zhu S., Yuan Y., Wang Y., Zeng L., Batley J., Wang Y.P. (2019). Transcriptomic comparison between developing seeds of yellow-and black-seeded *Brassica napus* reveals that genes influence seed quality. BMC Plant Biol..

[23] Ding L.N., Guo X.J., Li M., Fu Z.L., Yan S.Z., Zhu K.M., Wang Z., Tan X.L. (2019). Improving seed germination and oil contents by regulating the *GDSL* transcriptional level in *Brassica napus*. Plant Cell Rep..

[24] Xiao Z., Zhang C., Tang F., Yang B., Zhang L., Liu J., Huo Q., Wang S., Li S., Wei L. (2019). Identification of candidate genes controlling oil content by combination of genome-wide association and transcriptome analysis in the oilseed crop *Brassica napus*. Biotechnol. Biofuels.

[25] Qu C., Yin N., Chen S., Wang S., Chen X., Zhao H., Shen S., Fu F., Zhou B., Xu X. (2020). Comparative Analysis of the Metabolic Profiles of Yellow-versus Black-Seeded Rapeseed Using UPLC–HESI–MS/MS and Transcriptome Analysis. J. Agric. Food Chem..

[26] Niu Y., Wu L., Li Y., Huang H., Qian M., Sun W., Zhu H., Xu Y., Fan Y., Mahmood U. (2020). Deciphering the transcriptional regulatory networks that control size, color, and oil content in *Brassica rapa* seeds. Biotechnol. Biofuels.

[27] Lu K., Wei L., Li X., Wang Y., Wu J., Liu M., Zhang C., Chen Z., Xiao Z., Jian H. (2019). Whole-genome resequencing reveals *Brassica napus* origin and genetic loci involved in its improvement. Nat. Commun..

[28] Klees S., Lange T.M., Bertram H., Rajavel A., Schlüter J.-S., Lu K., Schmitt A.O., Gültas M. (2020). In Silico Identification of the Complex Interplay between Regulatory SNPs, Transcription Factors, and Their Related Genes in *Brassica napus* L. Using Multi-Omics Data. Int. J. Mol. Sci..

[29] Kaufmann K., Wellmer F., Muiño J.M., Ferrier T., Wuest S.E., Kumar V., Serrano-Mislata A., Madueno F., Krajewski P., Meyerowitz E.M. (2010). Orchestration of floral initiation by APETALA1. Science.

[30] Gómez-Mena C., de Folter S., Costa M.M.R., Angenent G.C., Sablowski R. (2005). Transcriptional program controlled by the floral homeotic gene AGAMOUS during early organogenesis. Development.

[31] Meyerowitz E.M. (2002). Plants compared to animals: The broadest comparative study of development. Science.

[32] Yanagisawa S. (1998). Transcription factors in plants: Physiological functions and regulation of expression. J. Plant Res..

[33] Singh K.B. (1998). Transcriptional regulation in plants: The importance of combinatorial control. Plant Physiol..

[34] Guilfoyle T.J. (1997). The structure of plant gene promoters. Genetic Engineering.

[35] Kaufmann K., Pajoro A., Angenent G.C. (2010). Regulation of transcription in plants: Mechanisms controlling developmental switches. Nat. Rev. Genet..

[36] Rajavel A., Heinrich F., Schmitt A.O., Gültas M. (2020). Identifying Cattle Breed-Specific Partner Choice of Transcription Factors during the African Trypanosomiasis Disease Progression Using Bioinformatics Analysis. Vaccines.

[37] Yi X., Du Z., Su Z. (2013). PlantGSEA: A gene set enrichment analysis toolkit for plant community. Nucleic Acids Res..

[38] Kambhampati S., Aznar-Moreno J.A., Hostetler C., Caso T., Bailey S.R., Hubbard A.H., Durrett T.P., Allen D.K. (2020). On the Inverse Correlation of Protein and Oil: Examining the Effects of Altered Central Carbon Metabolism on Seed Composition Using Soybean Fast Neutron Mutants. Metabolites.

[39] Rapala-Kozik M., Wolak N., Kujda M., Banas A.K. (2012). The upregulation of thiamine (vitamin B 1) biosynthesis in *Arabidopsis thaliana* seedlings under salt and osmotic stress conditions is mediated by abscisic acid at the early stages of this stress response. BMC Plant Biol..

[40] Steuernagel L., Meckbach C., Heinrich F., Zeidler S., Schmitt A.O., Gültas M. (2019). Computational identification of tissue-specific transcription factor cooperation in ten cattle tissues. PLoS ONE.

[41] Meckbach C., Wingender E., Gültas M. (2018). Removing background co-occurrences of transcription factor binding sites greatly improves the prediction of specific transcription factor cooperations. Front. Genet..

[42] Meckbach C., Tacke R., Hua X., Waack S., Wingender E., Gültas M. (2015). PC-TraFF: Identification of potentially collaborating transcription factors using pointwise mutual information. BMC Bioinform..

[43] Zeidler S., Meckbach C., Tacke R., Raad F.S., Roa A., Uchida S., Zimmermann W.H., Wingender E., Gültas M. (2016). Computational Detection of Stage-Specific Transcription Factor Clusters during Heart Development. Front. Genet..

[44] Collinge M., Boller T. (2001). Differential induction of two potato genes, *St*prx2 and *St*NAC, in response to infection by *Phytophthora infestans* and to wounding. Plant Mol. Biol..

[45] Hegedus D., Yu M., Baldwin D., Gruber M., Sharpe A., Parkin I., Whitwill S., Lydiate D. (2003). Molecular characterization of *Brassica napus* NAC domain transcriptional activators induced in response to biotic and abiotic stress. Plant Mol. Biol..

[46] Tran L.S.P., Nakashima K., Sakuma Y., Simpson S.D., Fujita Y., Maruyama K., Fujita M., Seki M., Shinozaki K., Yamaguchi-Shinozaki K. (2004). Isolation and functional analysis of Arabidopsis stress-inducible NAC transcription factors that bind to a drought-responsive cis-element in the early responsive to dehydration stress 1 promoter. Plant Cell.

[47] Fujita M., Fujita Y., Maruyama K., Seki M., Hiratsu K., Ohme-Takagi M., Tran L.S.P., Yamaguchi-Shinozaki K., Shinozaki K. (2004). A dehydration-induced NAC protein, RD26, is involved in a novel ABA-dependent stress-signaling pathway. Plant J..

[48] Olsen A.N., Ernst H.A., Leggio L.L., Skriver K. (2005). NAC transcription factors: Structurally distinct, functionally diverse. Trends Plant Sci..

[49] Balazadeh S., Siddiqui H., Allu A.D., Matallana-Ramirez L.P., Caldana C., Mehrnia M., Zanor M.I., Köhler B., Mueller-Roeber B. (2010). A gene regulatory network controlled by the NAC transcription factor ANAC092/*At*NAC2/ORE1 during salt-promoted senescence. Plant J..

[50] Ning Y.Q., Ma Z.Y., Huang H.W., Mo H., Zhao T.t., Li L., Cai T., Chen S., Ma L., He X.J. (2015). Two novel NAC transcription factors regulate gene expression and flowering time by associating with the histone demethylase JMJ14. Nucleic Acids Res..

[51] Yates A.D., Achuthan P., Akanni W., Allen J., Allen J., Alvarez-Jarreta J., Amode M.R., Armean I.M., Azov A.G., Bennett R. (2020). Ensembl 2020. Nucleic Acids Res..

[52] Bolser D.M., Staines D.M., Perry E., Kersey P.J. (2017). Ensembl plants: Integrating tools for visualizing, mining, and analyzing plant genomic data. Plant Genomics Databases.

[53] Argüello-Astorga G., Herrera-Estrella L. (1998). Evolution of light-regulated plant promoters. Annu. Rev. Plant Biol..

[54] Koch K. (1996). Carbohydrate-modulated gene expression in plants. Annu. Rev. Plant Biol..

[55] Terzaghi W.B., Cashmore A.R. (1995). Photomorphenesis: Seeing the light in plant development. Curr. Biol..

[56] Rastogi R., Bate N.J., Sivasankar S., Rothstein S.J. (1997). Footprinting of the spinach nitrite reductase gene promoter reveals the preservation of nitrate regulatory elements between fungi and higher plants. Plant Mol. Biol..

[57] Oliveira I.C., Coruzzi G.M. (1999). Carbon and amino acids reciprocally modulate the expression of glutamine synthetase in Arabidopsis. Plant Physiol..

[58] Yeap W.C., Lee F.C., Shabari Shan D.K., Musa H., Appleton D.R., Kulaveerasingam H. (2017). WRI 1-1, ABI 5, NF-YA 3 and NF-YC 2 increase oil biosynthesis in coordination with hormonal signaling during fruit development in oil palm. Plant J..

[59] Huang X., Hou L., Meng J., You H., Li Z., Gong Z., Yang S., Shi Y. (2018). The antagonistic action of abscisic acid and cytokinin signaling mediates drought stress response in Arabidopsis. Mol. Plant.

[60] Yanagisawa S. (2002). The Dof family of plant transcription factors. Trends Plant Sci..

[61] Ahmad M., Rim Y., Chen H., Kim J. (2013). Functional characterization of Arabidopsis Dof transcription factor *At*Dof4. 1. Russ. J. Plant Physiol..

[62] Santopolo S., Boccaccini A., Lorrai R., Ruta V., Capauto D., Minutello E., Serino G., Costantino P., Vittorioso P. (2015). *DOF AFFECTING GERMINATION 2* is a positive regulator of light-mediated seed germination and is repressed by *DOF AFFECTING GERMINATION 1*. BMC Plant Biol..

[63] Wang H.W., Zhang B., Hao Y.J., Huang J., Tian A.G., Liao Y., Zhang J.S., Chen S.Y. (2007). The soybean Dof-type transcription factor genes, *Gm*Dof4 and *Gm*Dof11, enhance lipid content in the seeds of transgenic Arabidopsis plants. Plant J..

[64] Fobert P.R. (2012). Role of Transcription Factors in Storage Lipid Accumulation in Plants.

[65] Su Y., Liang W., Liu Z., Wang Y., Zhao Y., Ijaz B., Hua J. (2017). Overexpression of *Gh*Dof1 improved salt and cold tolerance and seed oil content in Gossypium hirsutum. J. Plant Physiol..

[66] Rerie W.G., Feldmann K.A., Marks M.D. (1994). The GLABRA2 gene encodes a homeo domain protein required for normal trichome development in Arabidopsis. Genes Dev..

[67] Shen B., Sinkevicius K.W., Selinger D.A., Tarczynski M.C. (2006). The homeobox gene GLABRA2 affects seed oil content in Arabidopsis. Plant Mol. Biol..

[68] Ariel F.D., Manavella P.A., Dezar C.A., Chan R.L. (2007). The true story of the HD-Zip family. Trends Plant Sci..

[69] Kubo H., Peeters A.J., Aarts M.G., Pereira A., Koornneef M. (1999). *ANTHOCYANINLESS2*, a homeobox gene affecting anthocyanin distribution and root development in Arabidopsis. Plant Cell.

[70] Yu H., Chen X., Hong Y.Y., Wang Y., Xu P., Ke S.D., Liu H.Y., Zhu J.K., Oliver D.J., Xiang C.B. (2008). Activated expression of an Arabidopsis HD-START protein confers drought tolerance with improved root system and reduced stomatal density. Plant Cell.

[71] Javelle M., Vernoud V., Depege-Fargeix N., Arnould C., Oursel D., Domergue F., Sarda X., Rogowsky P.M. (2010). Overexpression of the epidermis-specific homeodomain-leucine zipper IV transcription factor Outer Cell Layer1 in maize identifies target genes involved in lipid metabolism and cuticle biosynthesis. Plant Physiol..

[72] Grimberg Å., Lager I., Street N.R., Robinson K.M., Marttila S., Mähler N., Ingvarsson P.K., Bhalerao R.P. (2018). Storage lipid accumulation is controlled by photoperiodic signal acting via regulators of growth cessation and dormancy in hybrid aspen. New Phytol..

[73] Lechner E., Leonhardt N., Eisler H., Parmentier Y., Alioua M., Jacquet H., Leung J., Genschik P. (2011). MATH/BTB CRL3 receptors target the homeodomain-leucine zipper ATHB6 to modulate abscisic acid signaling. Dev. Cell.

[74] Leung J., Merlot S., Giraudat J. (1997). The Arabidopsis *ABSCISIC ACID-INSENSITIVE2* (*ABI2*) and *ABI1* genes encode homologous protein phosphatases 2C involved in abscisic acid signal transduction. Plant Cell.

[75] Son O., Cho H.Y., Kim M.R., Lee H., Lee M.S., Song E., Park J.H., Nam K.H., Chun J.Y., Kim H.J. (2004). Induction of a homeodomain–leucine zipper gene by auxin is inhibited by cytokinin in Arabidopsis roots. Biochem. Biophys. Res. Commun..

[76] Jin H., Martin C. (1999). Multifunctionality and diversity within the plant MYB-gene family. Plant Mol. Biol..

[77] Ambawat S., Sharma P., Yadav N.R., Yadav R.C. (2013). MYB transcription factor genes as regulators for plant responses: An overview. Physiol. Mol. Biol. Plants.

[78] Mu R.L., Cao Y.R., Liu Y.F., Lei G., Zou H.F., Liao Y., Wang H.W., Zhang W.K., Ma B., Du J.Z. (2009). An R2R3-type transcription factor gene *At*MYB59 regulates root growth and cell cycle progression in Arabidopsis. Cell Res..

[79] Liao Y., Zou H.F., Wang H.W., Zhang W.K., Ma B., Zhang J.S., Chen S.Y. (2008). Soybean *Gm*MYB76, *Gm*MYB92, and *Gm*MYB177 genes confer stress tolerance in transgenic Arabidopsis plants. Cell Res..

[80] Araki S., Ito M., Soyano T., Nishihama R., Machida Y. (2004). Mitotic cyclins stimulate the activity of c-Myb-like factors for transactivation of G2/M phase-specific genes in tobacco. J. Biol. Chem..

[81] Schwinn K.E., Ngo H., Kenel F., Brummell D.A., Albert N.W., McCallum J.A., Pither-Joyce M., Crowhurst R.N., Eady C., Davies K.M. (2016). The onion (*Allium cepa* L.) R2R3-MYB gene MYB1 regulates anthocyanin biosynthesis. Front. Plant Sci..

[82] Chiu L.W., Li L. (2012). Characterization of the regulatory network of *Bo*MYB2 in controlling anthocyanin biosynthesis in purple cauliflower. Planta.

[83] Medina-Puche L., Molina-Hidalgo F.J., Boersma M., Schuurink R.C., López-Vidriero I., Solano R., Franco-Zorrilla J.M., Caballero J.L., Blanco-Portales R., Muñoz-Blanco J. (2015). An R2R3-MYB transcription factor regulates eugenol production in ripe strawberry fruit receptacles. Plant Physiol..

[84] Wang X.C., Wu J., Guan M.L., Zhao C.H., Geng P., Zhao Q. (2020). Arabidopsis MYB4 plays dual roles in flavonoid biosynthesis. Plant J..

[85] Ko J.H., Jeon H.W., Kim W.C., Kim J.Y., Han K.H. (2014). The MYB46/MYB83-mediated transcriptional regulatory programme is a gatekeeper of secondary wall biosynthesis. Ann. Bot..

[86] Ramírez V., García-Andrade J., Vera P. (2011). Enhanced disease resistance to *Botrytis cinerea* in myb46 *Arabidopsis* plants is associated to an early down-regulation of CesA genes. Plant Signal. Behav..

[87] Kim Y., Park S., Gilmour S.J., Thomashow M.F. (2013). Roles of CAMTA transcription factors and salicylic acid in configuring the low-temperature transcriptome and freezing tolerance of A rabidopsis. Plant J..

[88] Doherty C.J., Van Buskirk H.A., Myers S.J., Thomashow M.F. (2009). Roles for *Arabidopsis* CAMTA transcription factors in cold-regulated gene expression and freezing tolerance. Plant Cell.

[89] Du L., Ali G.S., Simons K.A., Hou J., Yang T., Reddy A., Poovaiah B. (2009). Ca^2+^/calmodulin regulates salicylic-acid-mediated plant immunity. Nature.

[90] Kumar M., Le D.T., Hwang S., Seo P.J., Kim H.U. (2019). Role of the *INDETERMINATE DOMAIN* Genes in Plants. Int. J. Mol. Sci..

[91] Colasanti J., Tremblay R., Wong A.Y., Coneva V., Kozaki A., Mable B.K. (2006). The maize *INDETERMINATE1* flowering time regulator defines a highly conserved zinc finger protein family in higher plants. BMC Genom..

[92] Lu J., Xu Y., Wang J., Singer S.D., Chen G. (2020). The Role of Triacylglycerol in Plant Stress Response. Plants.

[93] Wang H.W., Sun H.J., Chang T.Y., Lo H.H., Cheng W.C., Tseng G.C., Lin C.T., Chang S.J., Pal N.R., Chung I.F. (2015). Discovering monotonic stemness marker genes from time-series stem cell microarray data. BMC Genom. BioMed Cent..

[94] Chalhoub B., Denoeud F., Liu S., Parkin I.A., Tang H., Wang X., Chiquet J., Belcram H., Tong C., Samans B. (2014). Early allopolyploid evolution in the post-Neolithic *Brassica napus* oilseed genome. Science.

[95] Dobin A., Davis C.A., Schlesinger F., Drenkow J., Zaleski C., Jha S., Batut P., Chaisson M., Gingeras T.R. (2013). STAR: Ultrafast universal RNA-seq aligner. Bioinformatics.

[96] Anders S., Pyl P.T., Huber W. (2014). HTSeq—A Python framework to work with high-throughput sequencing data. bioRxiv.

[97] Smyth G.K., Ritchie M., Thorne N., Wettenhall J., Shi W., Hu Y. (2002). Limma: Linear Models for Microarray and RNA-Seq Data User’s Guide.

[98] Supek F., Bošnjak M., Škunca N., Šmuc T. (2011). REVIGO summarizes and visualizes long lists of gene ontology terms. PLoS ONE.

[99] Kel A.E., Gossling E., Reuter I., Cheremushkin E., Kel-Margoulis O.V., Wingender E. (2003). MATCHTM: A tool for searching transcription factor binding sites in DNA sequences. Nucleic Acids Res..

[100] Khan A., Fornes O., Stigliani A., Gheorghe M., Castro-Mondragon J.A., Van Der Lee R., Bessy A., Chèneby J., Kulkarni S.R., Tan G. (2018). JASPAR 2018: Update of the open-access database of transcription factor binding profiles and its web framework. Nucleic Acids Res..

