# Supplementary material for: Unravelling the Complex Interplay of Transcription Factors Orchestrating Seed Oil Content in Brassica napus L
Source: Int J Mol Sci. 2021 Jan 21;22(3):1033. doi: 10.3390/ijms22031033 (PMC7864344; doi:10.3390/ijms22031033)

Supplementary Information

Gene expression profiles of genes present in the cooperation networks for the seed tissue of Zhongshuang11 (ZS11) and Zhongyou821 (ZY821).

Visualisation of the expression values of transcription factor (TF) orthologous genes. The TFs and their significant cooperation have been presented in the manuscript in Figures 2 and 3. In the figure legends below, gene ID represents the Ensembl ID of the orthologous genes and gene symbols refer to the transcription factor gene names. In the following plots, y-axis represents the gene expression values and x-axis represents the time points 1, 2, 3 and 4 indicating day 7, day 10, day 15 and day 45 after flowering.

Figure S1

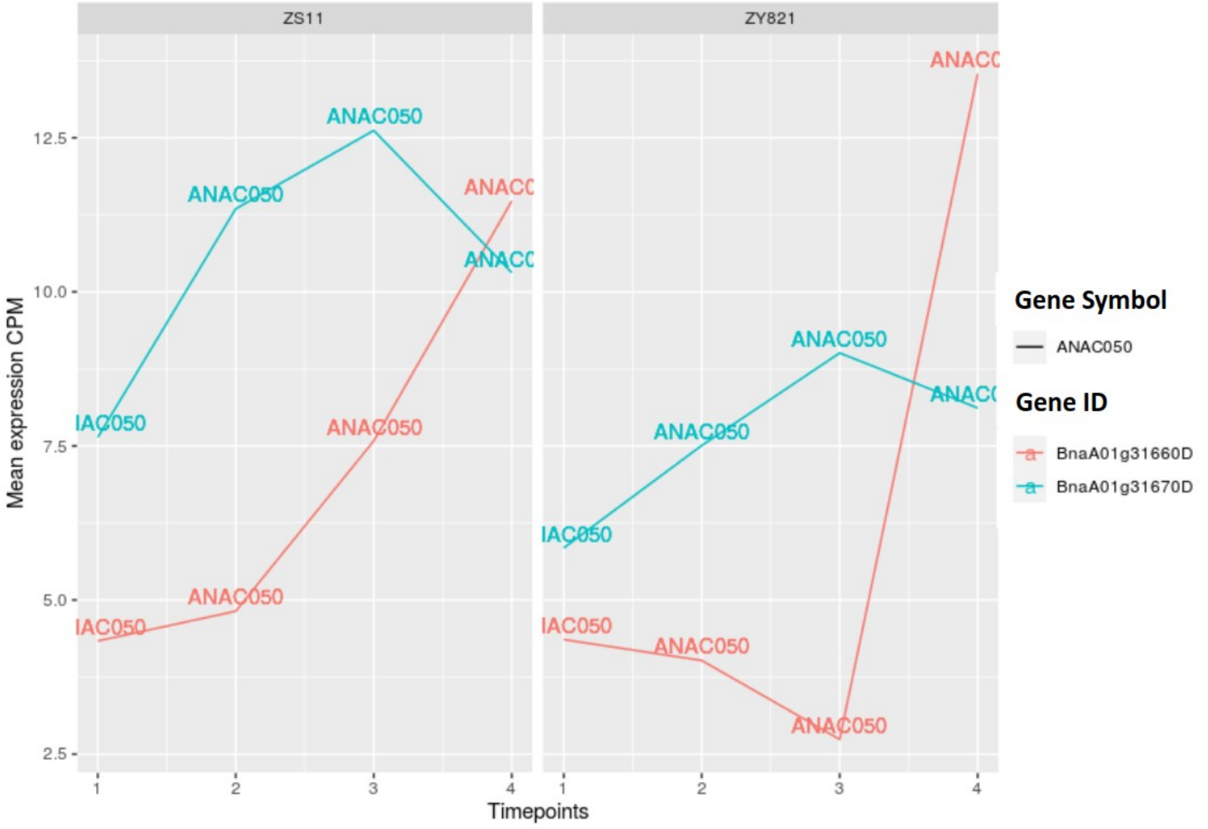

Figure S2

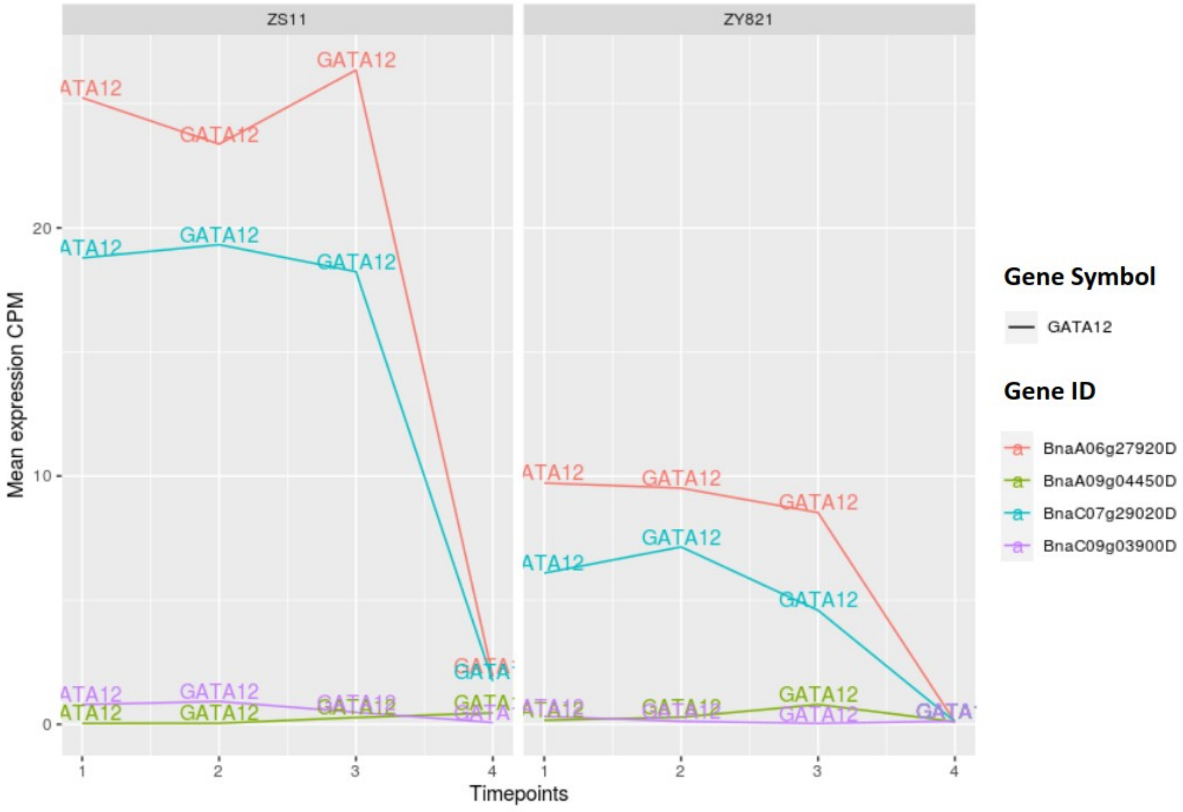

Figure S3

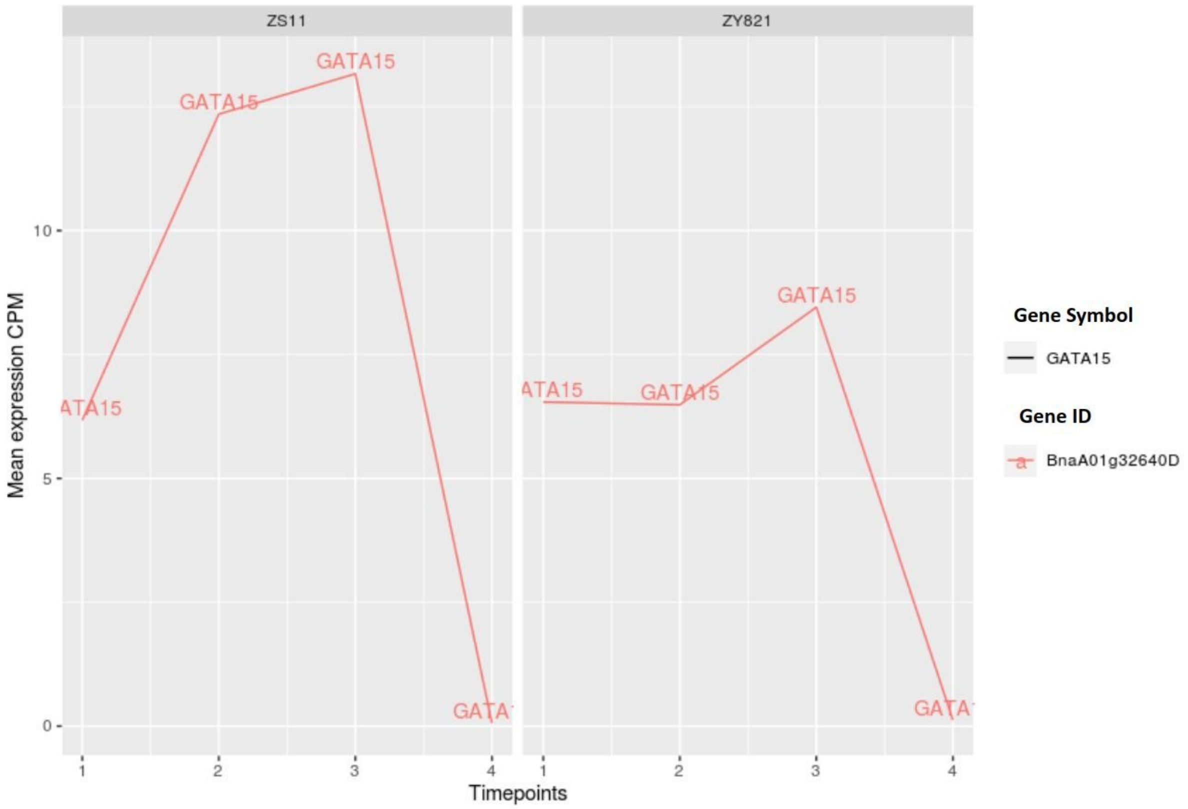

Figure S4

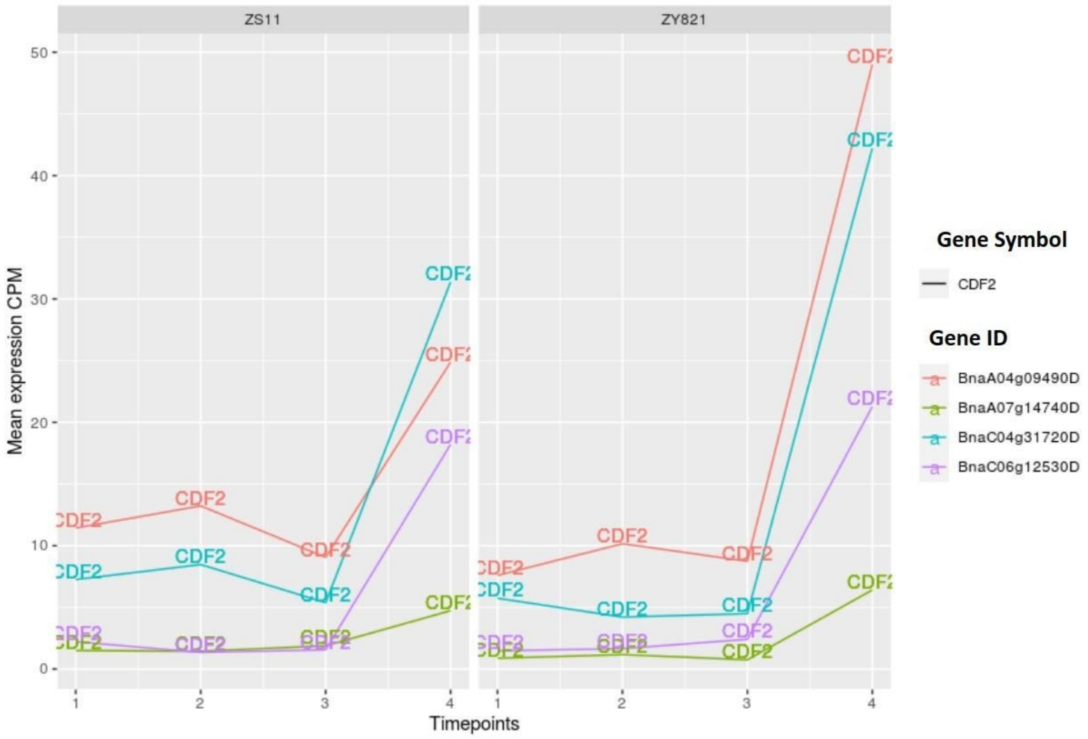

Figure S5

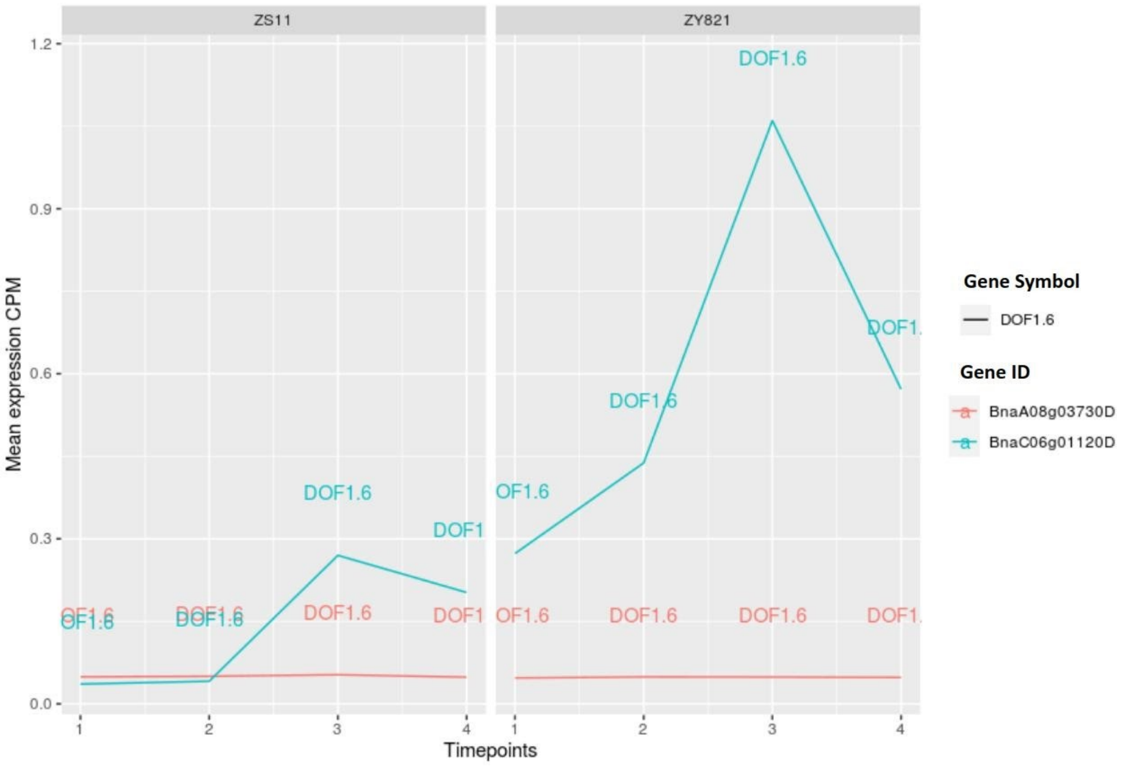

Figure S6

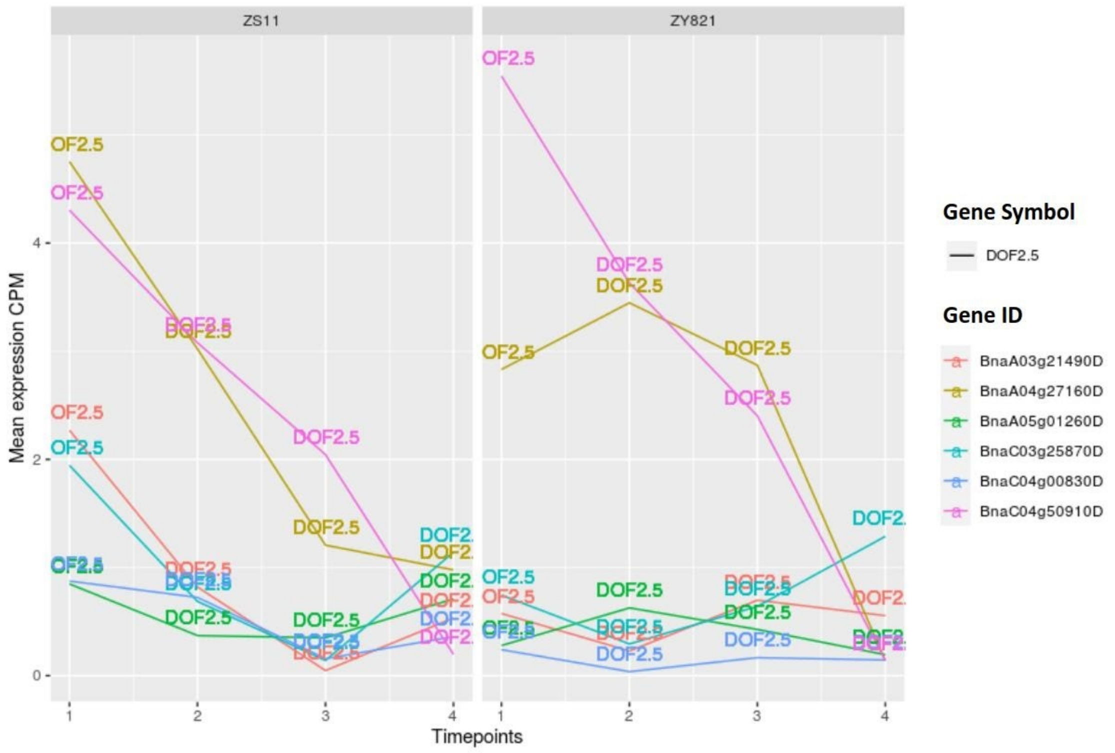

Figure S7

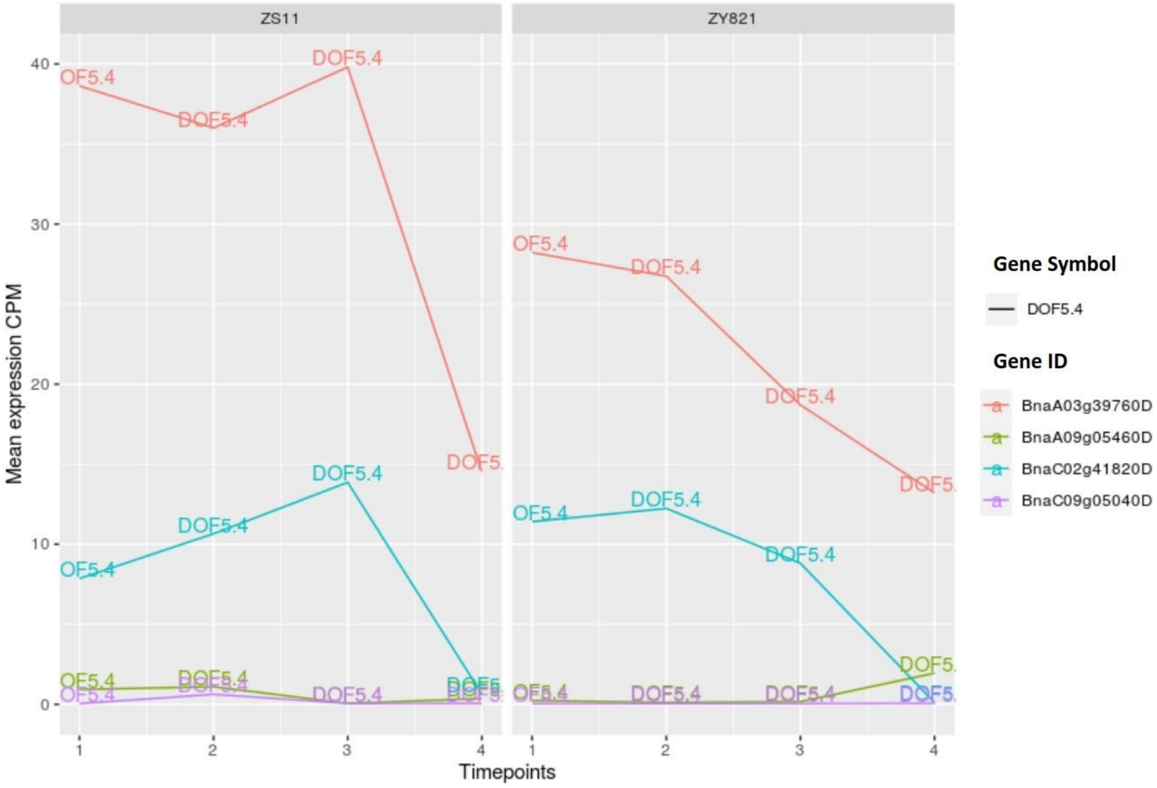

Figure S8

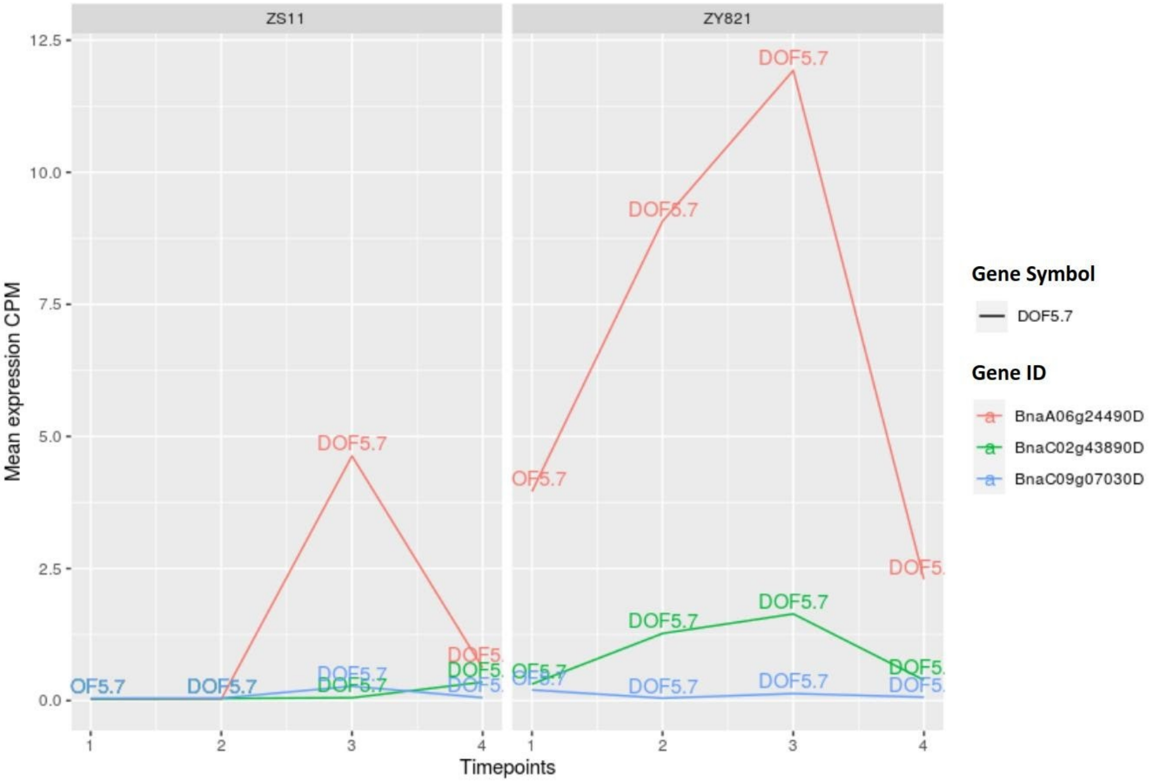

Figure S9

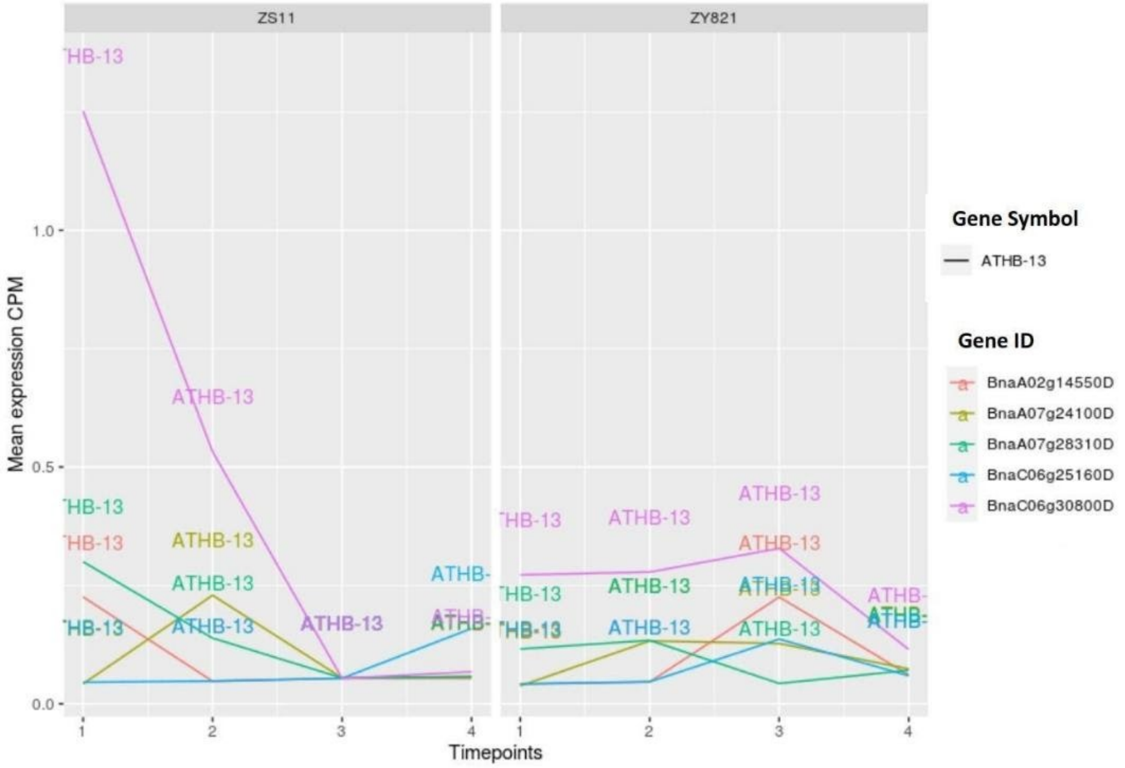

Figure S10

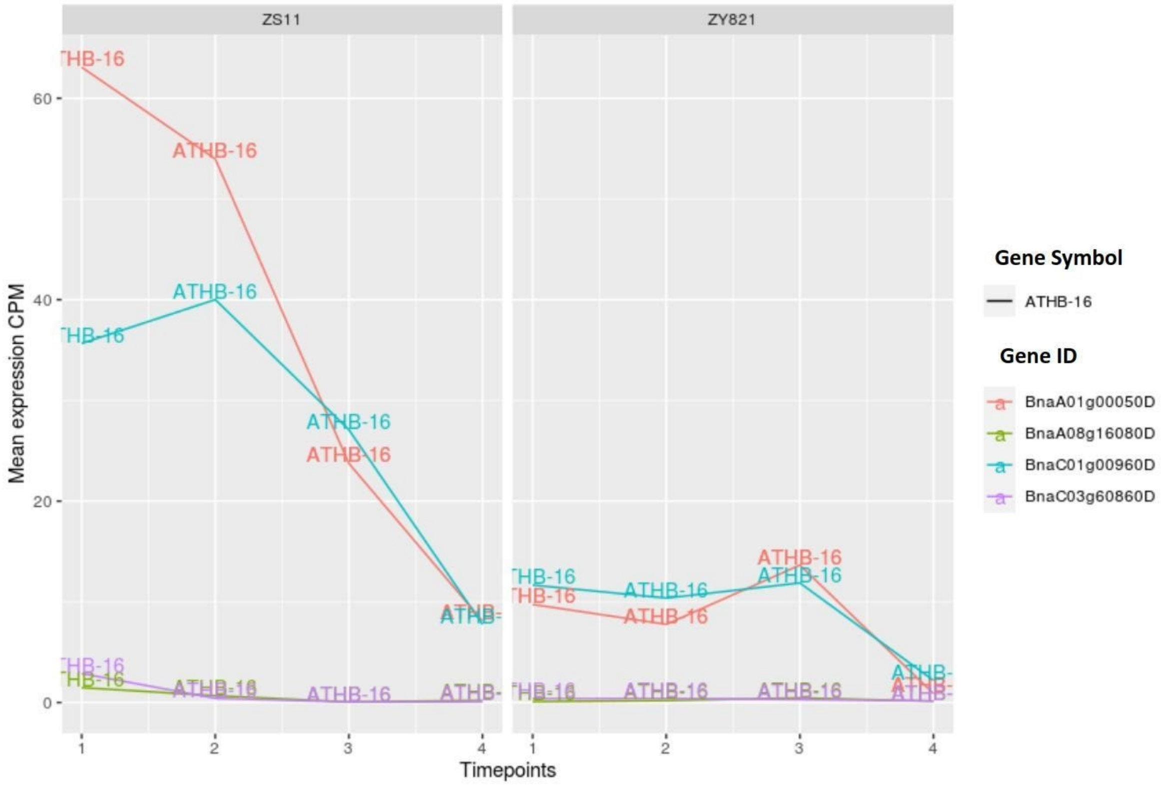

Figure S11

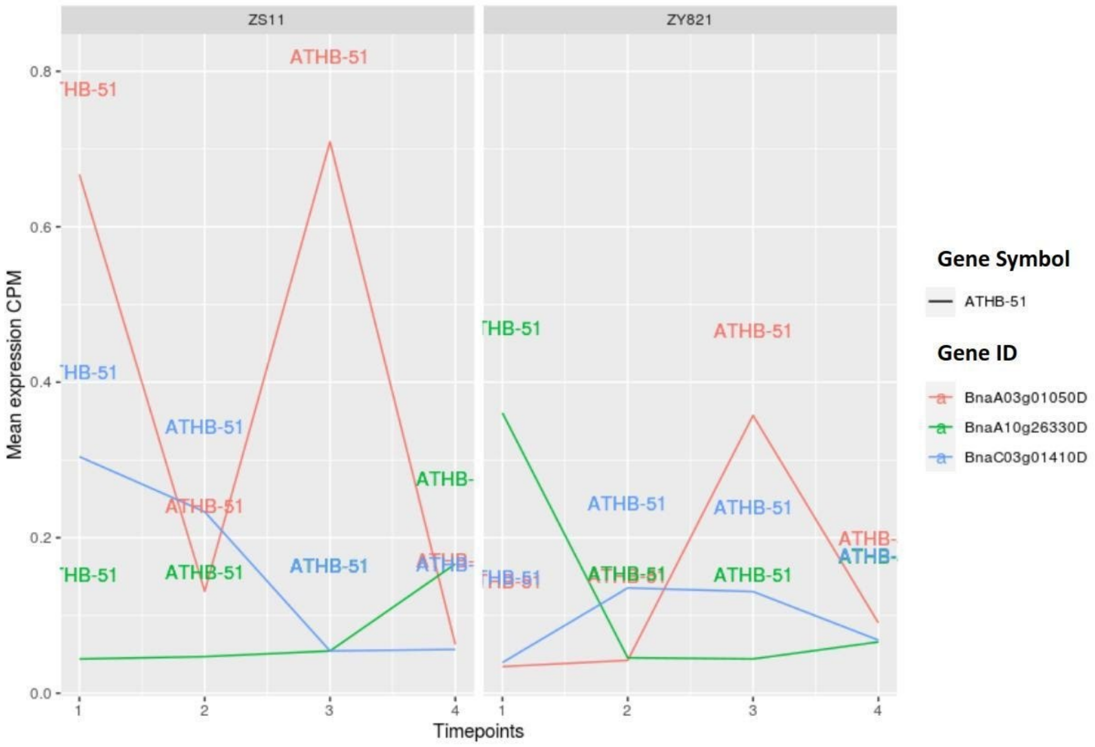

Figure S12

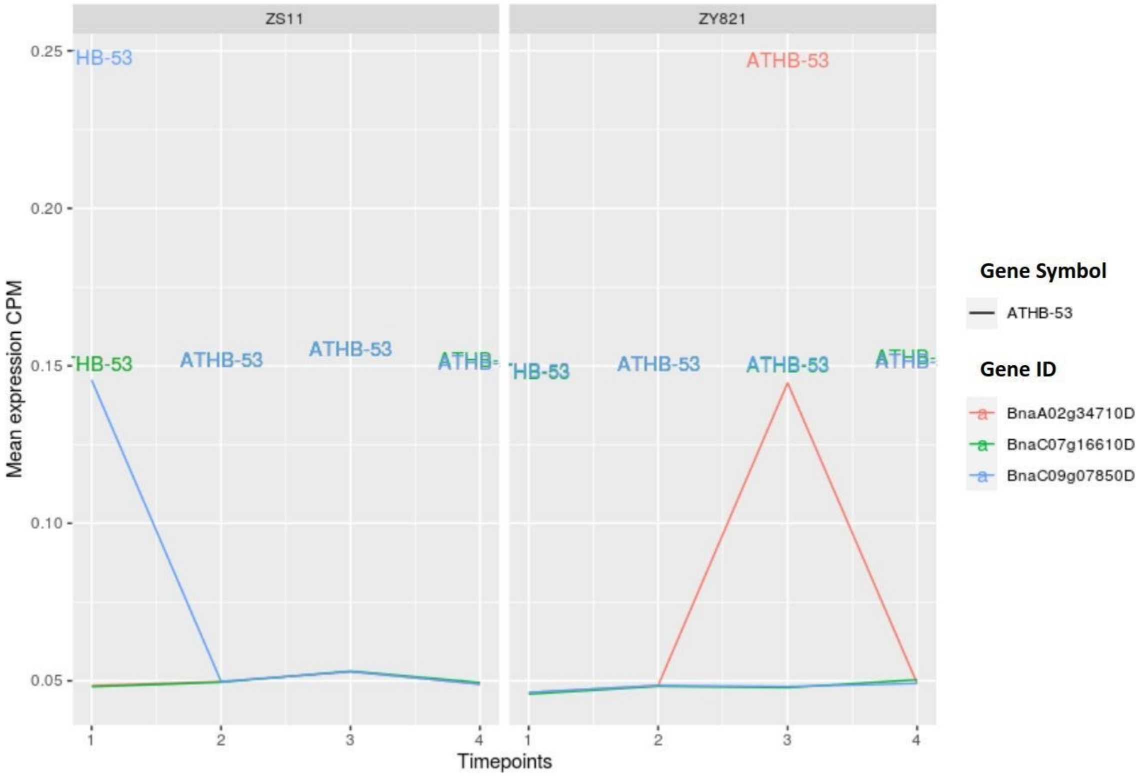

Figure S13

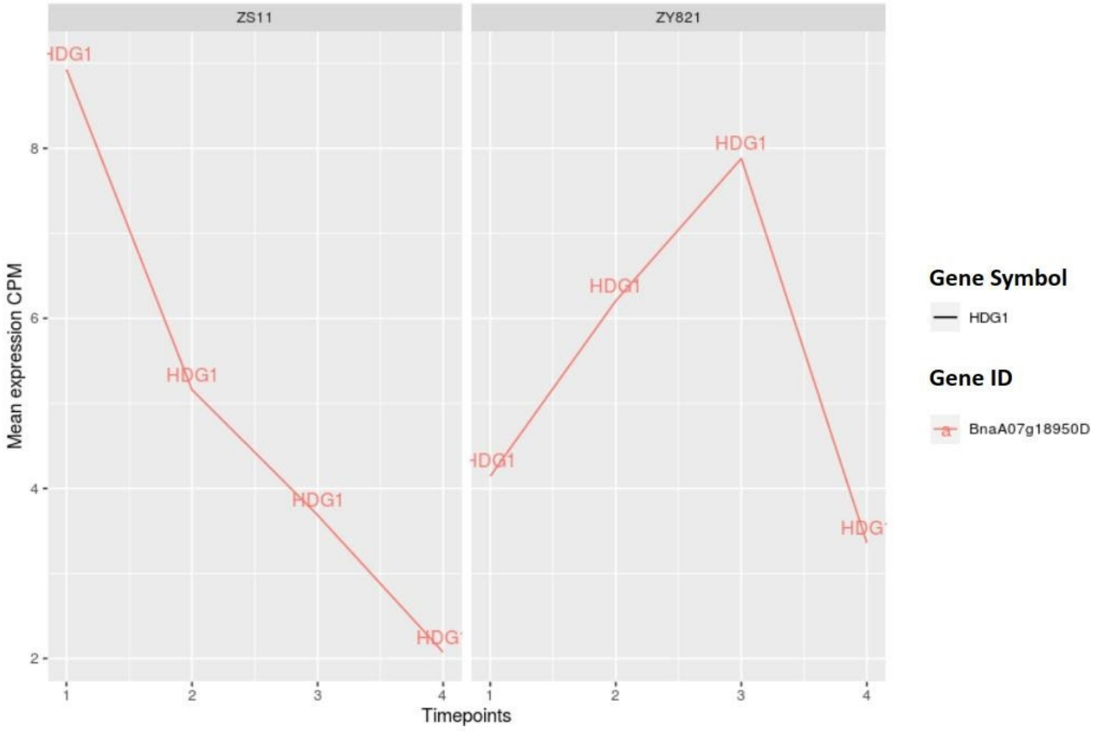

Figure S14

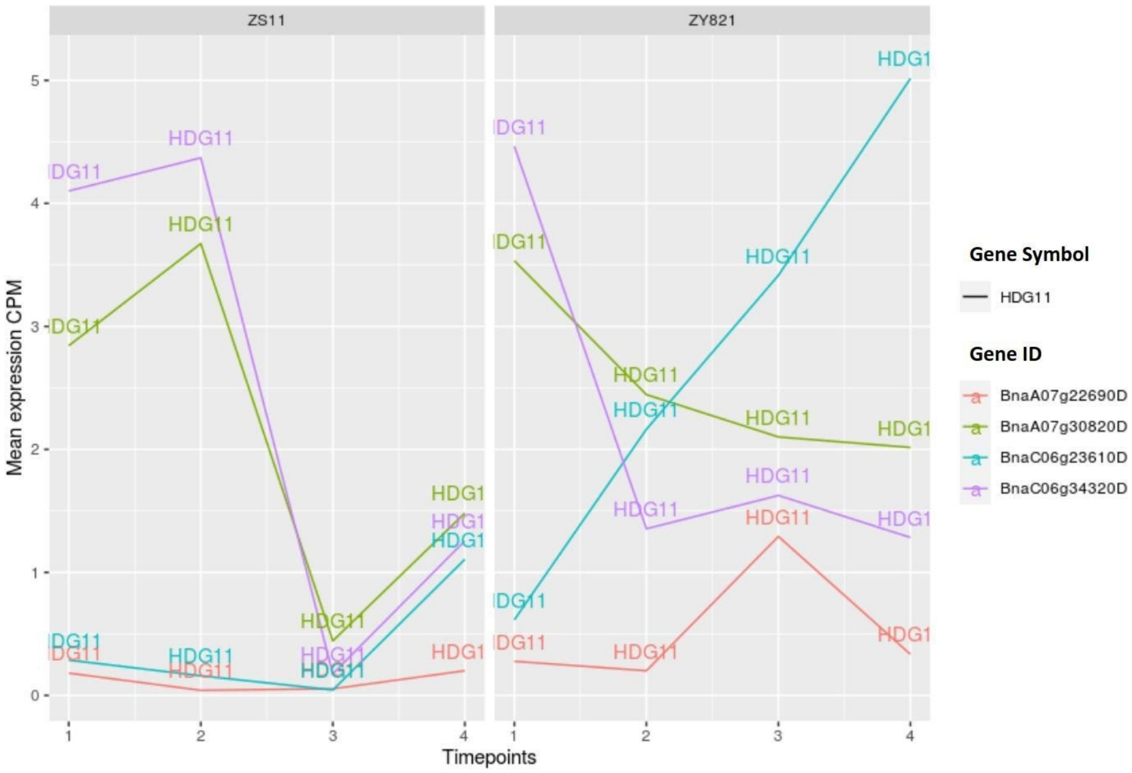

Figure S15

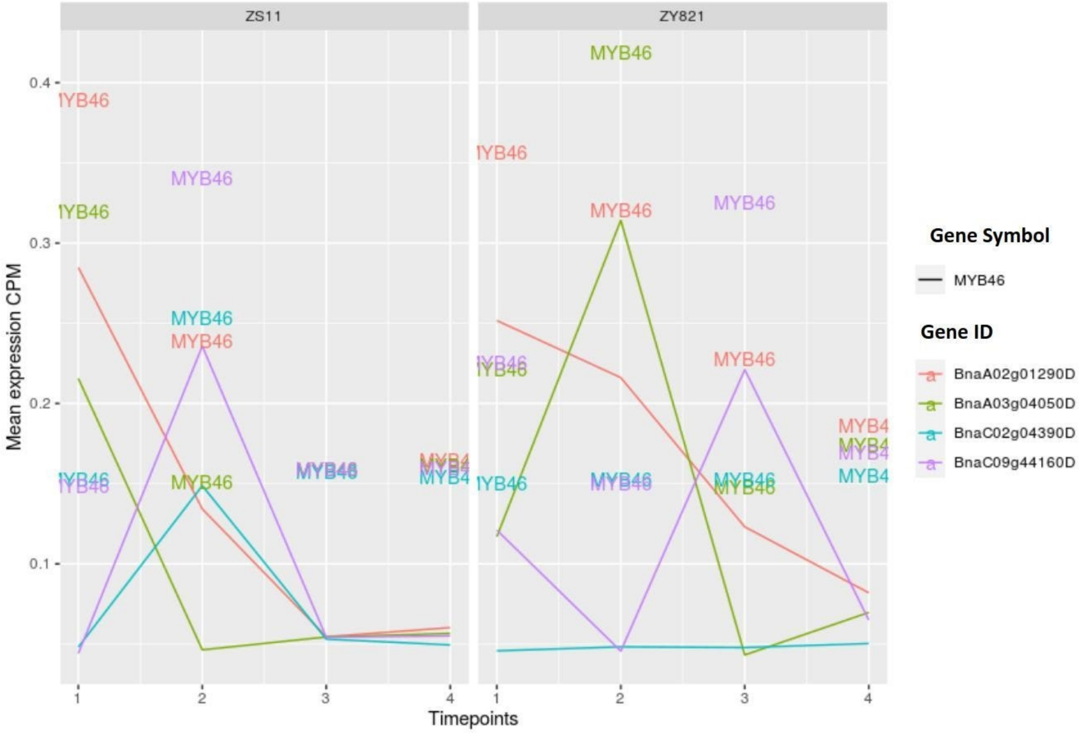

Figure S16

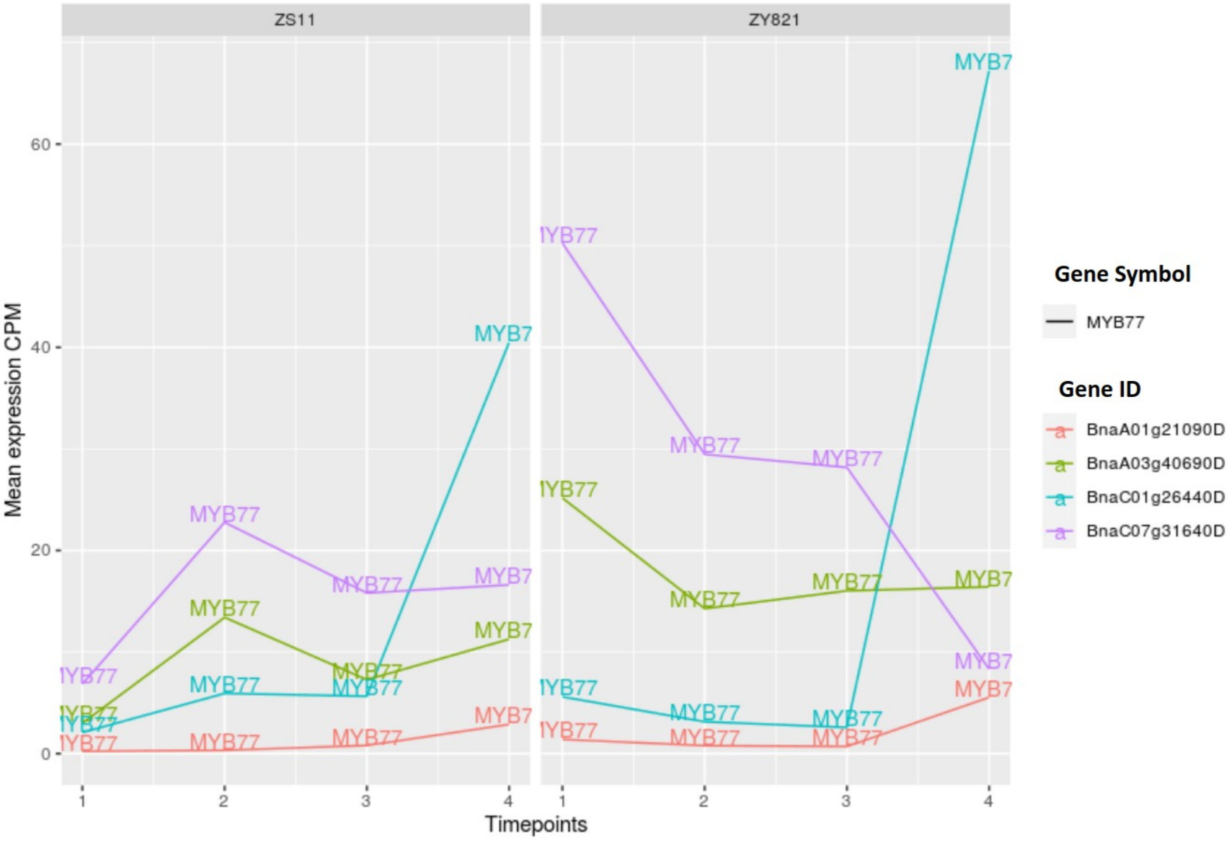

Figure S17

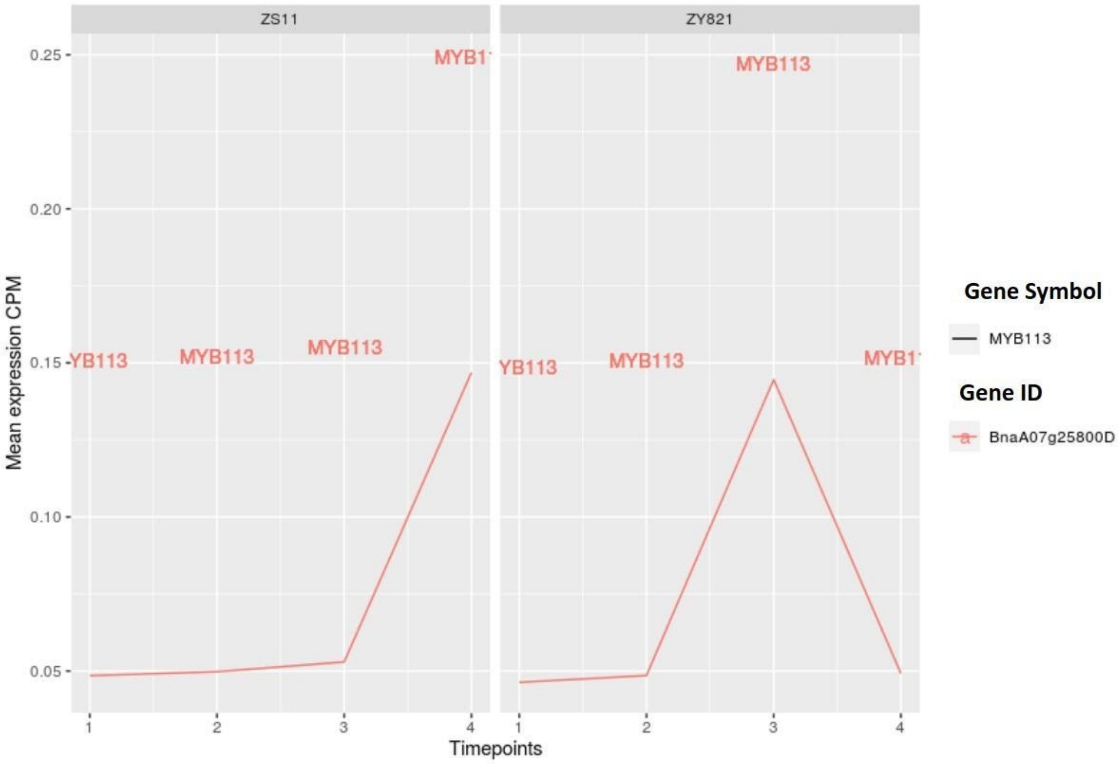

Figure S18

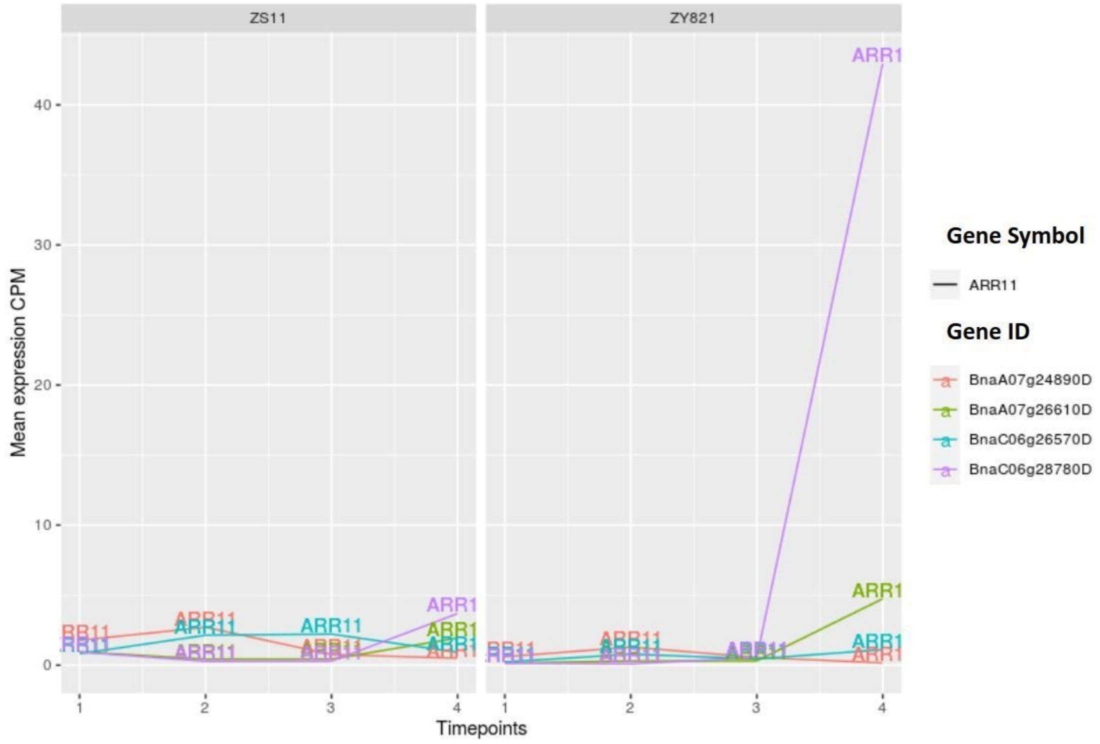

Figure S19

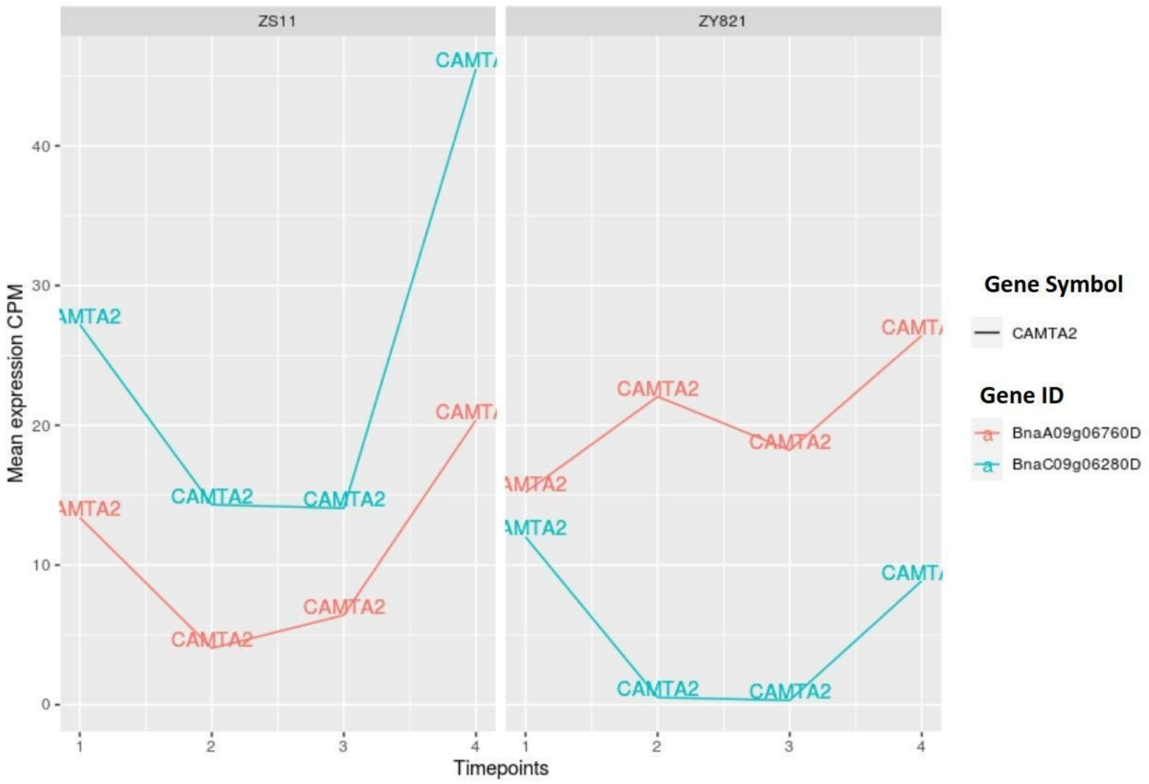

Figure S20

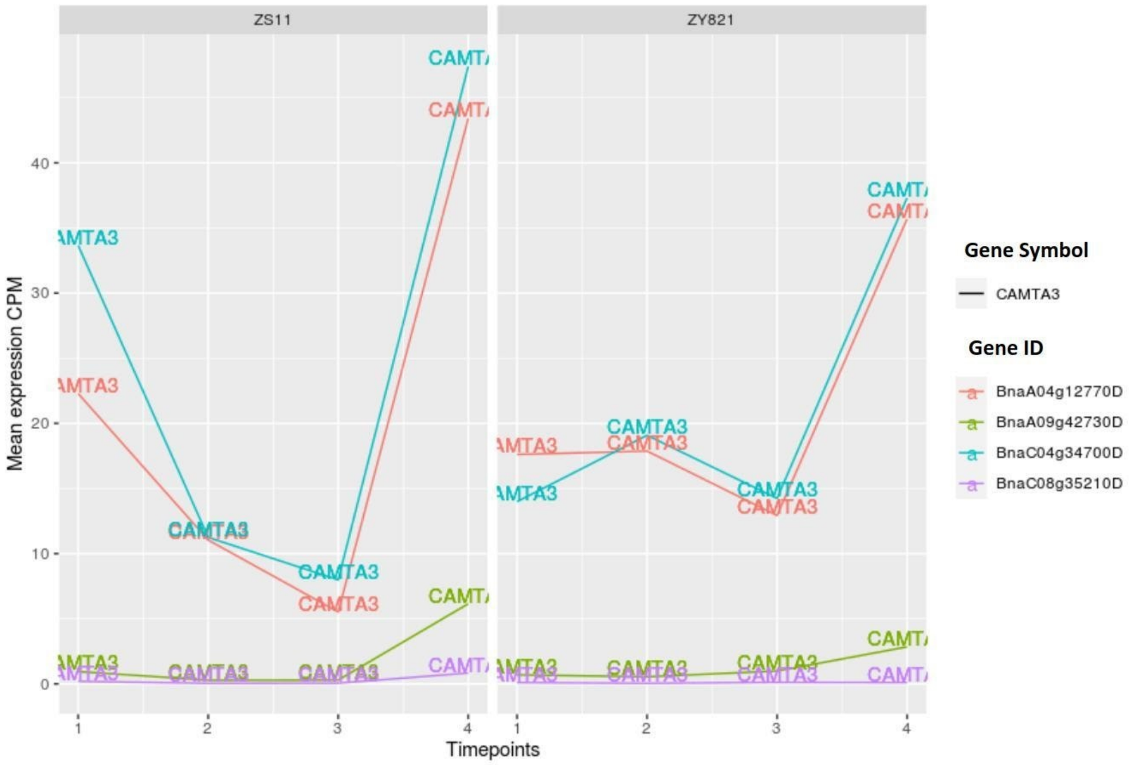

Figure S21

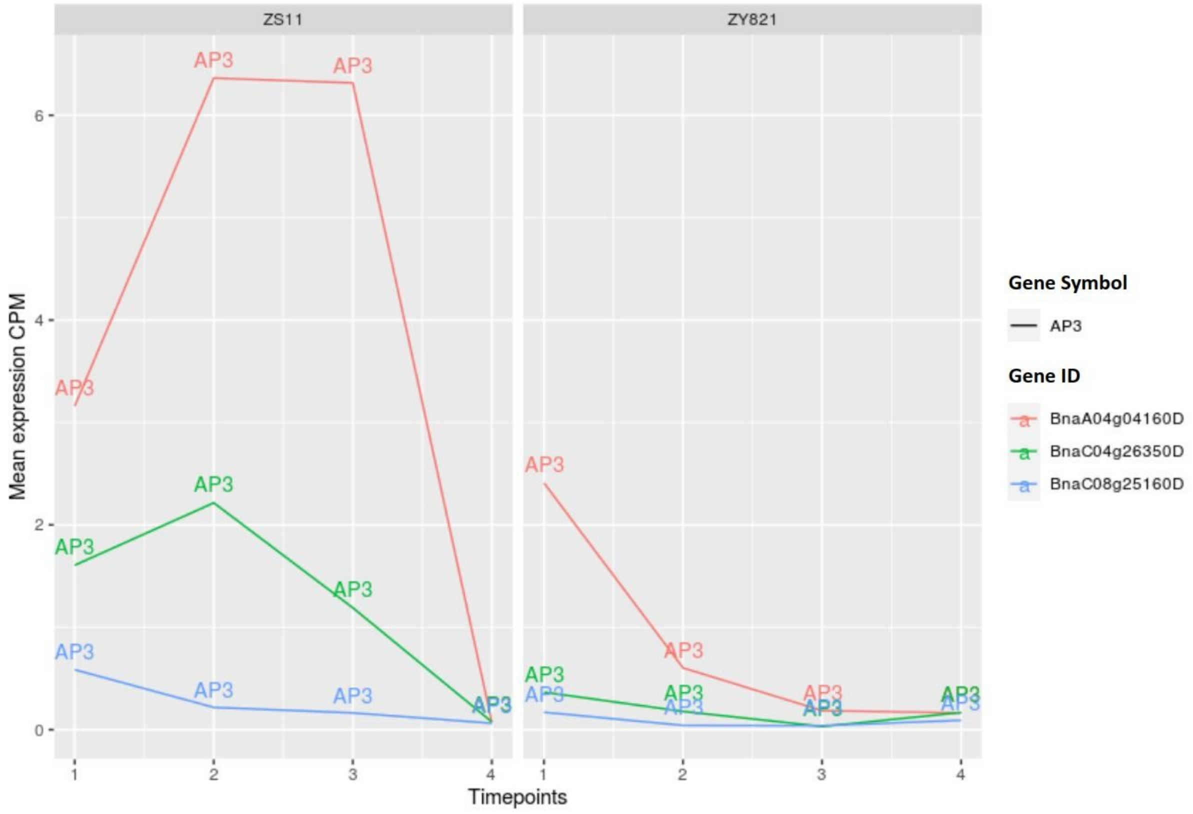

Figure S22

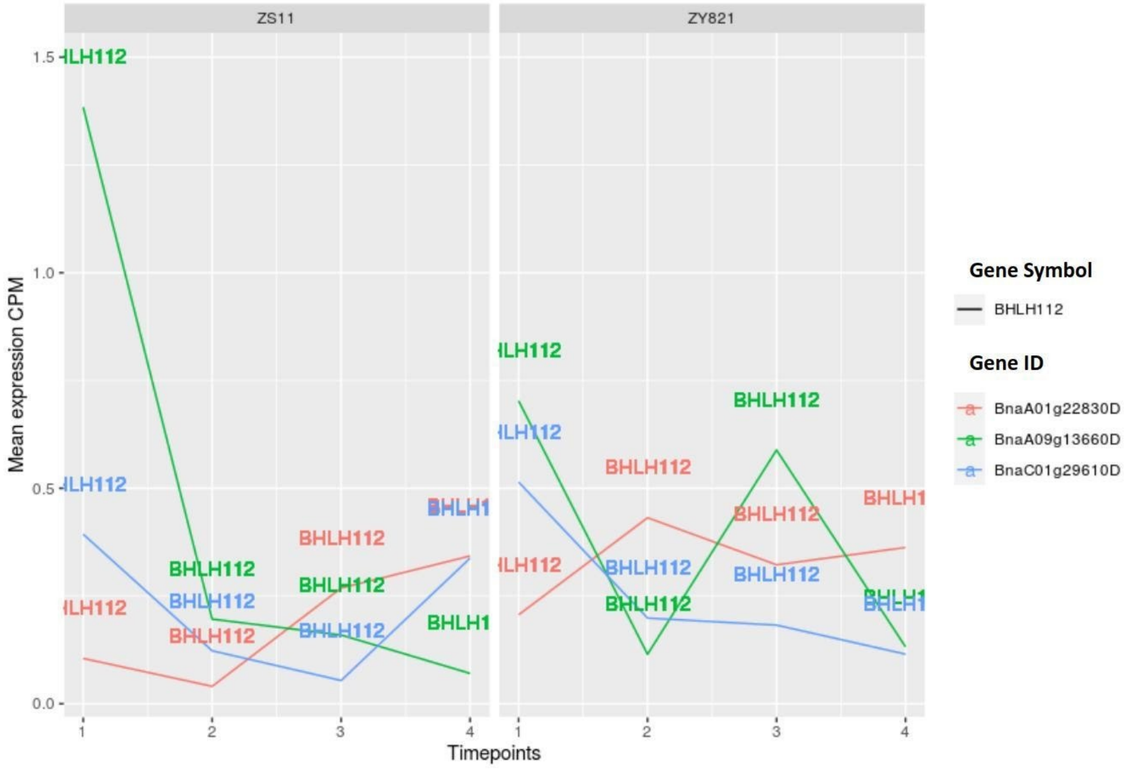

Figure S23

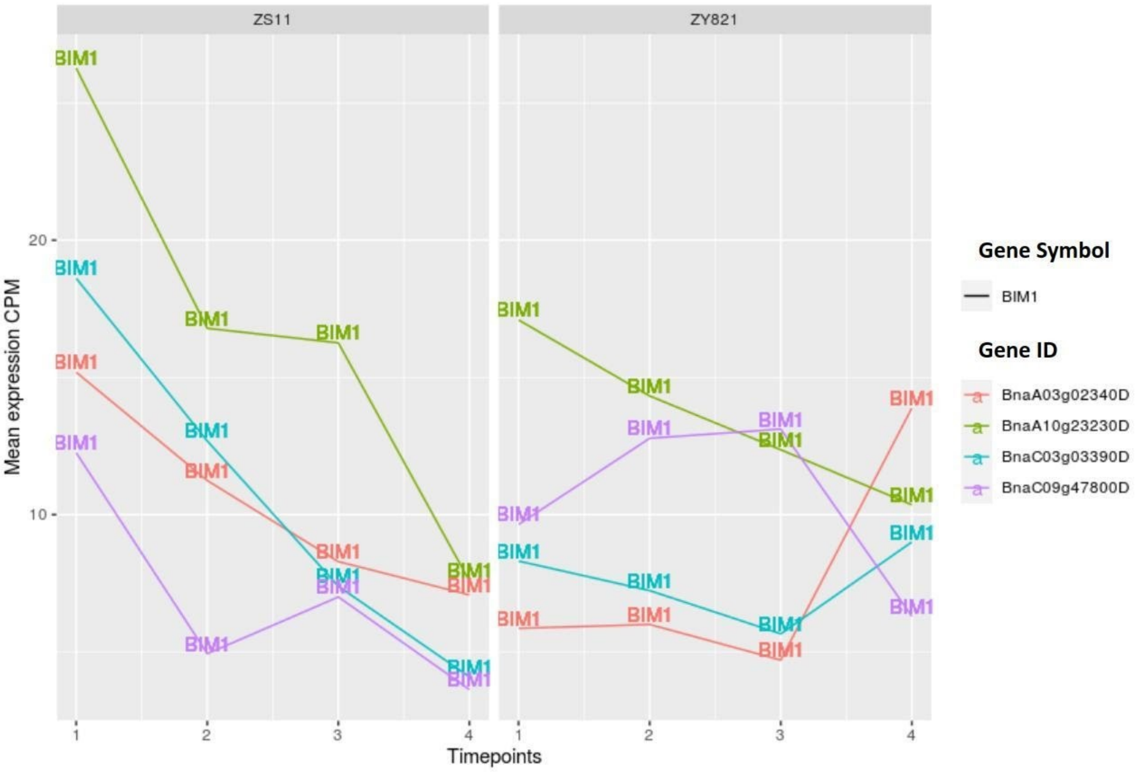

Figure S24

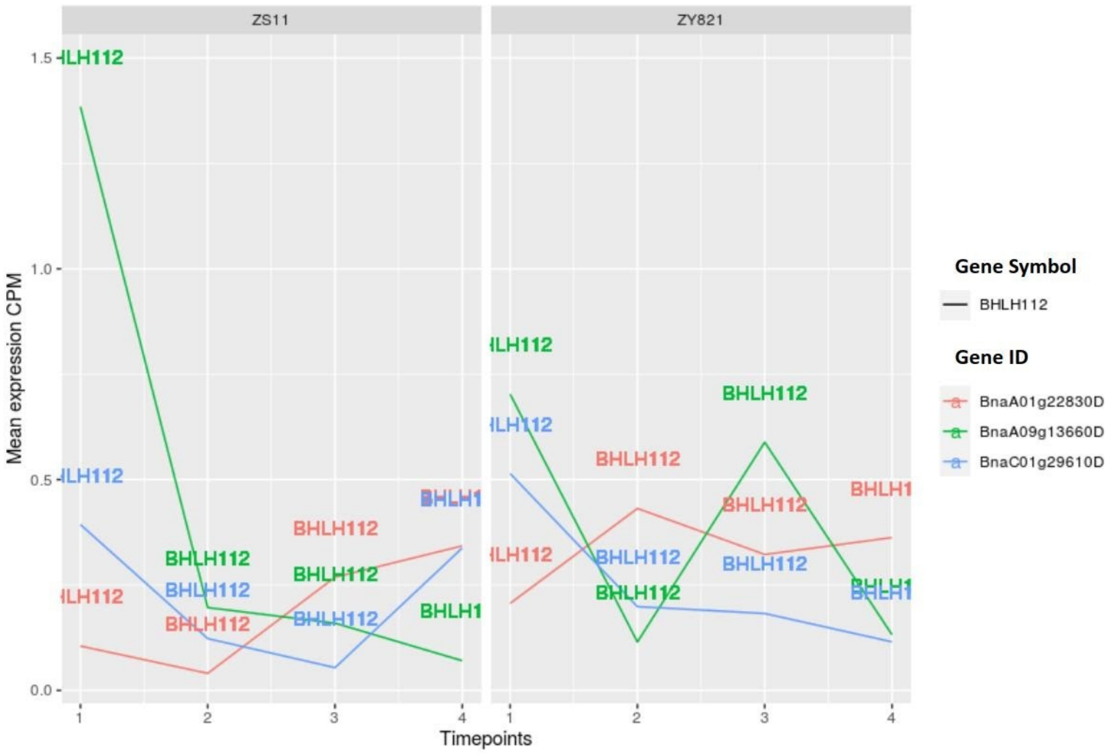

Figure S25

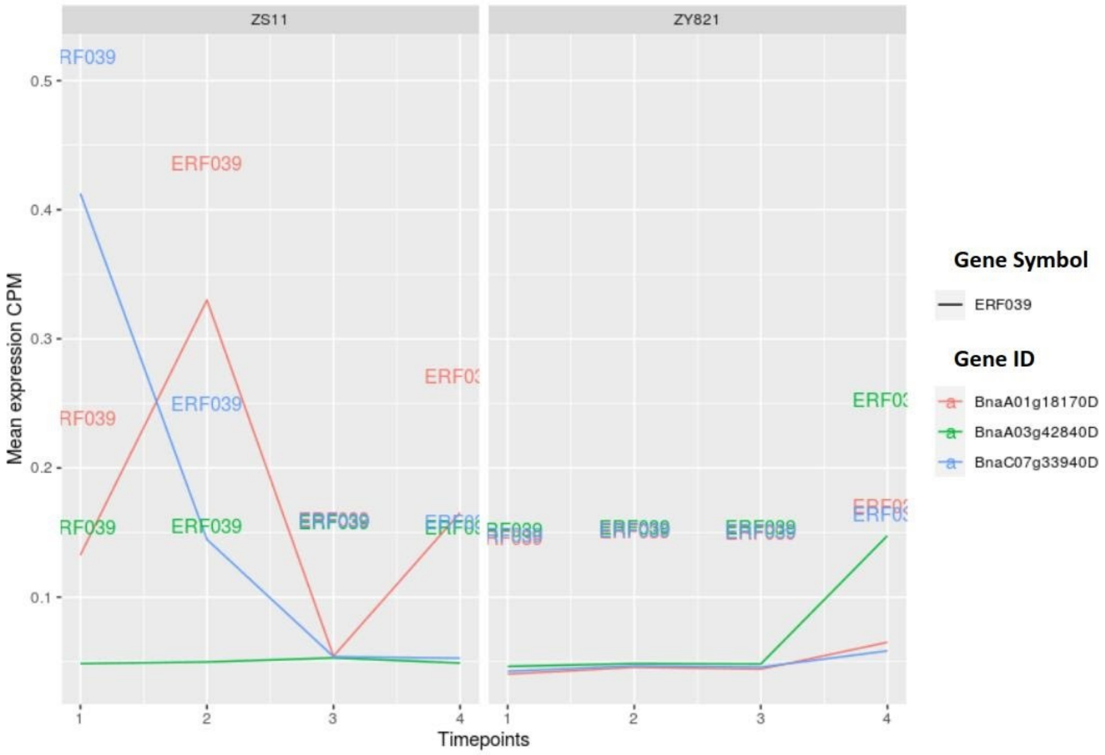

Figure S26

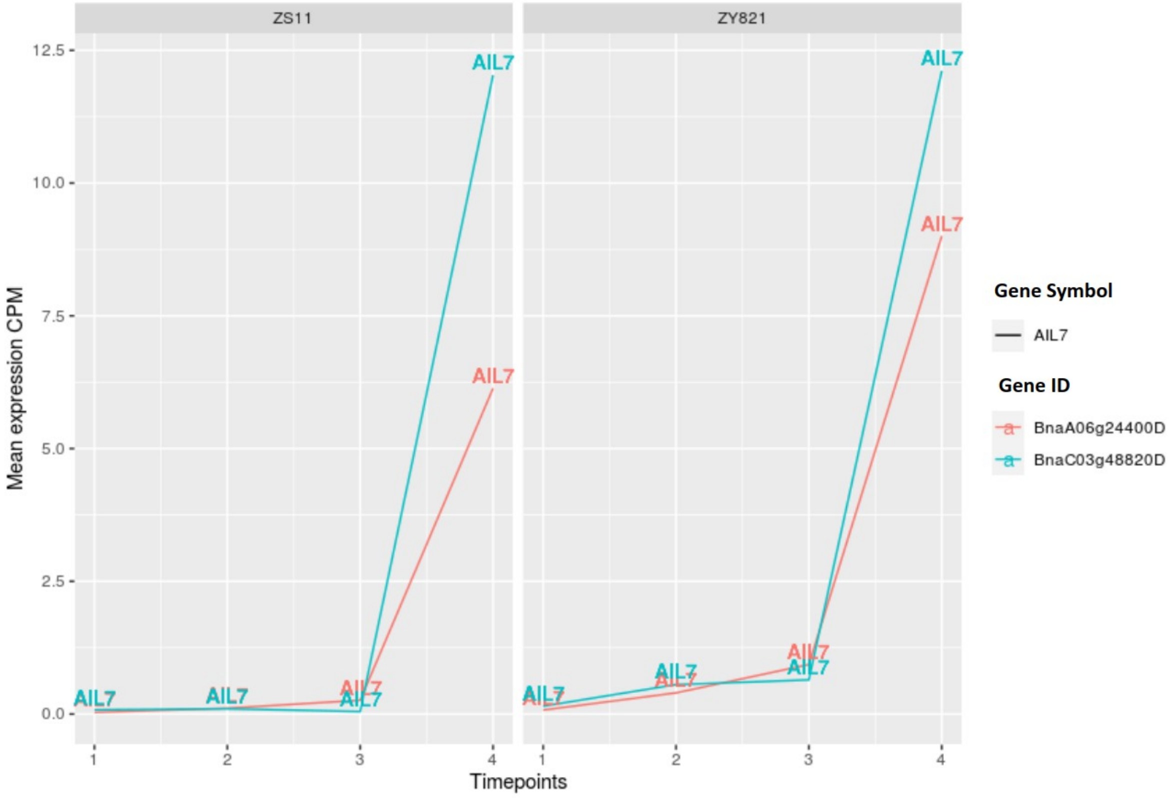

Figure S27

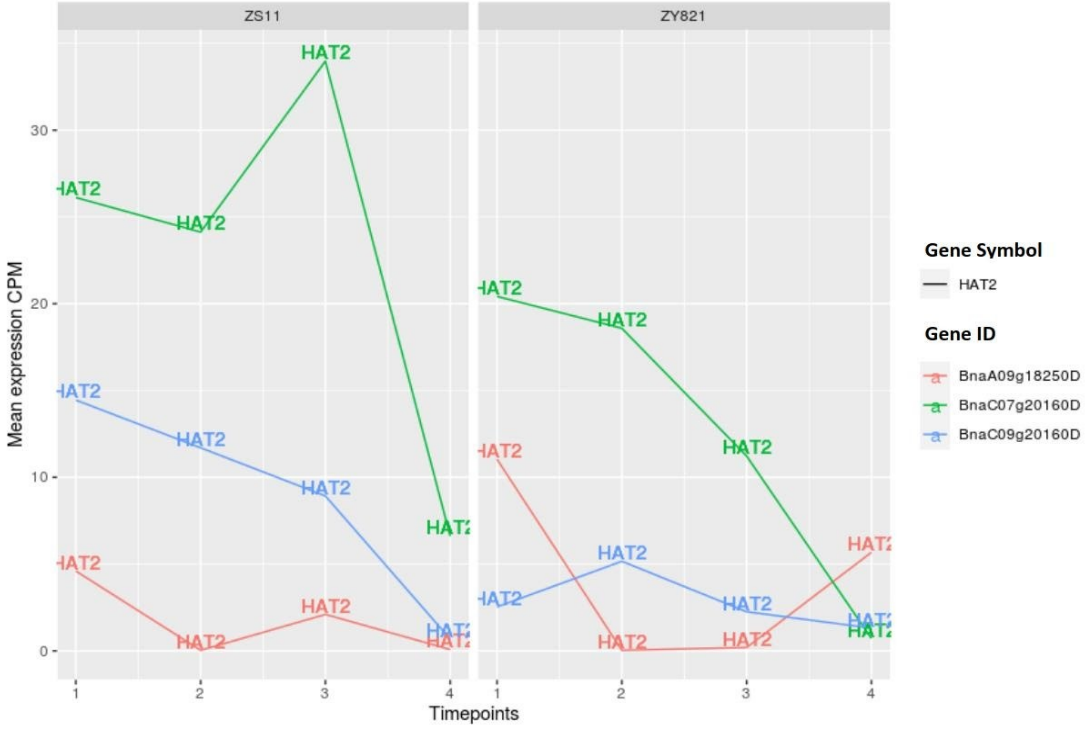

Figure S28

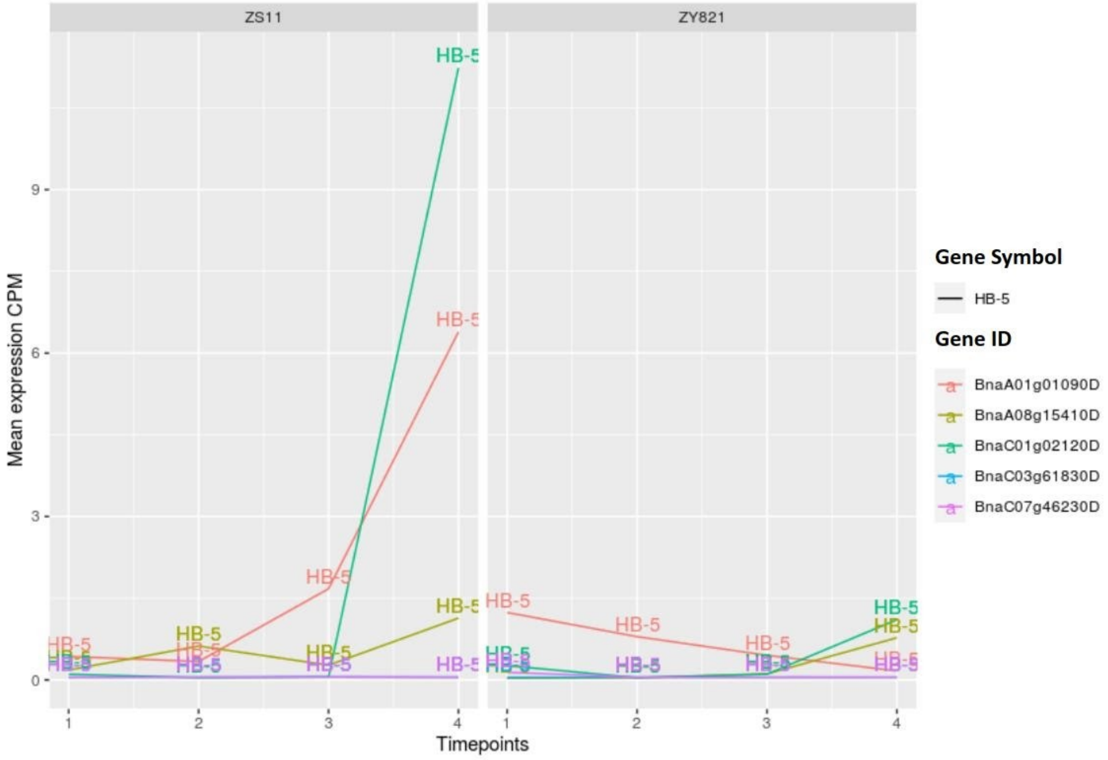

Figure S29

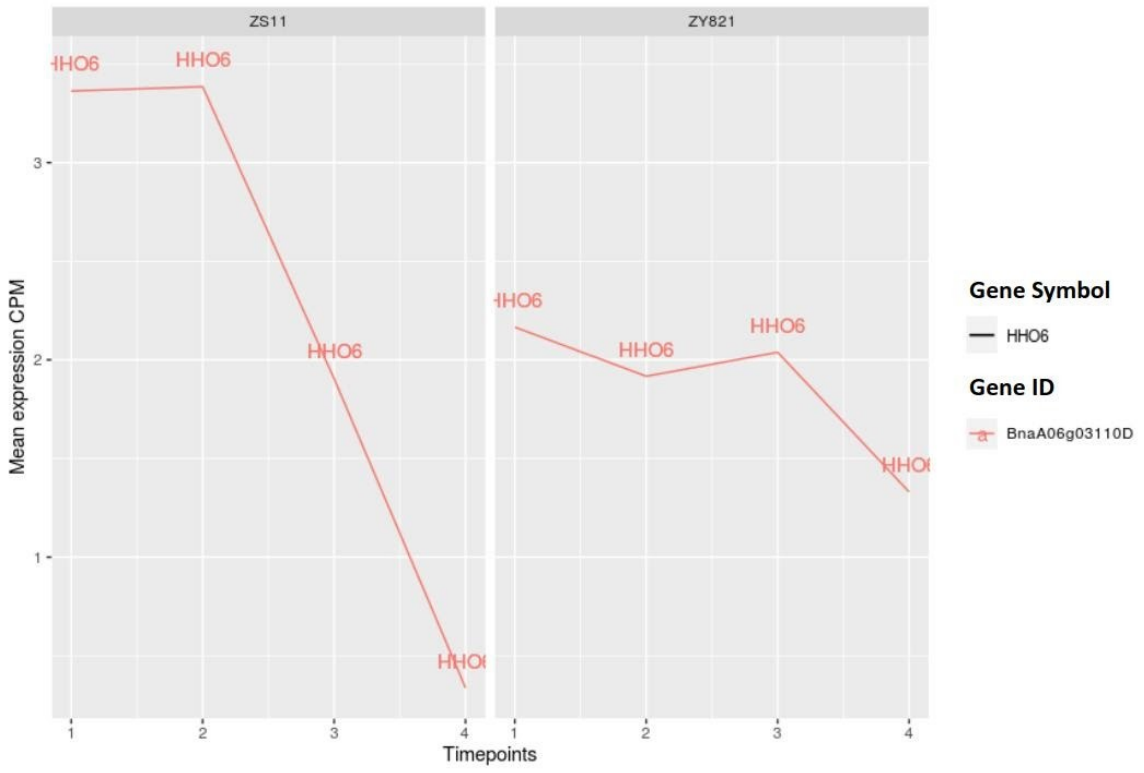

Figure S30

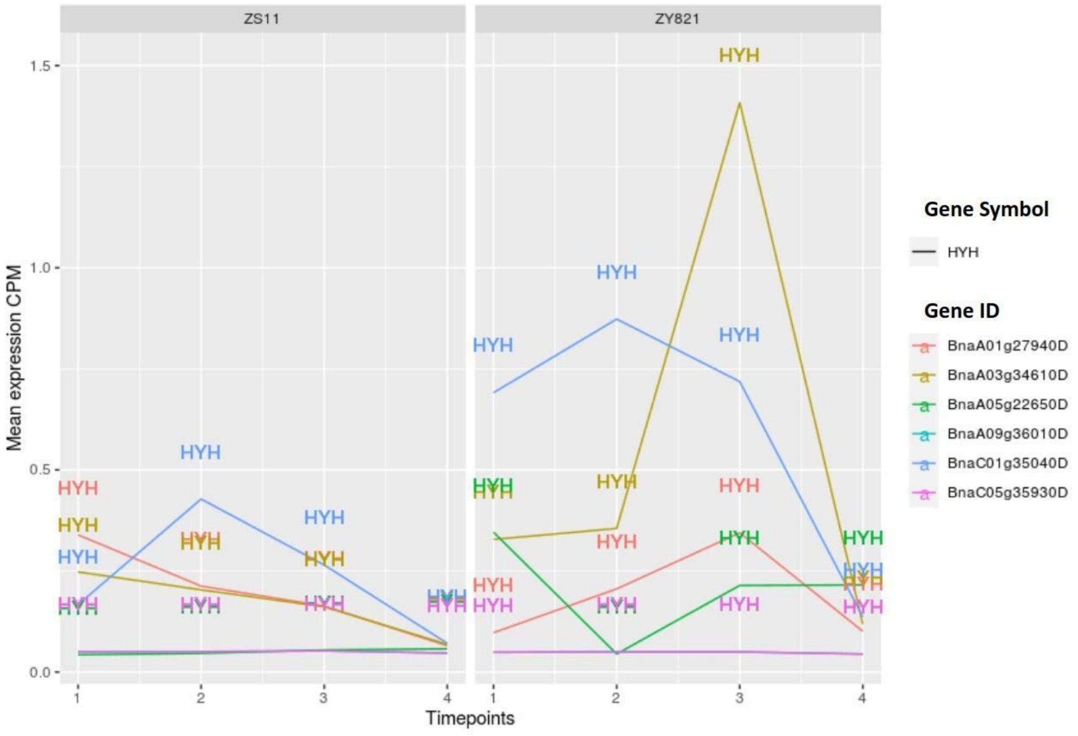

Figure S31

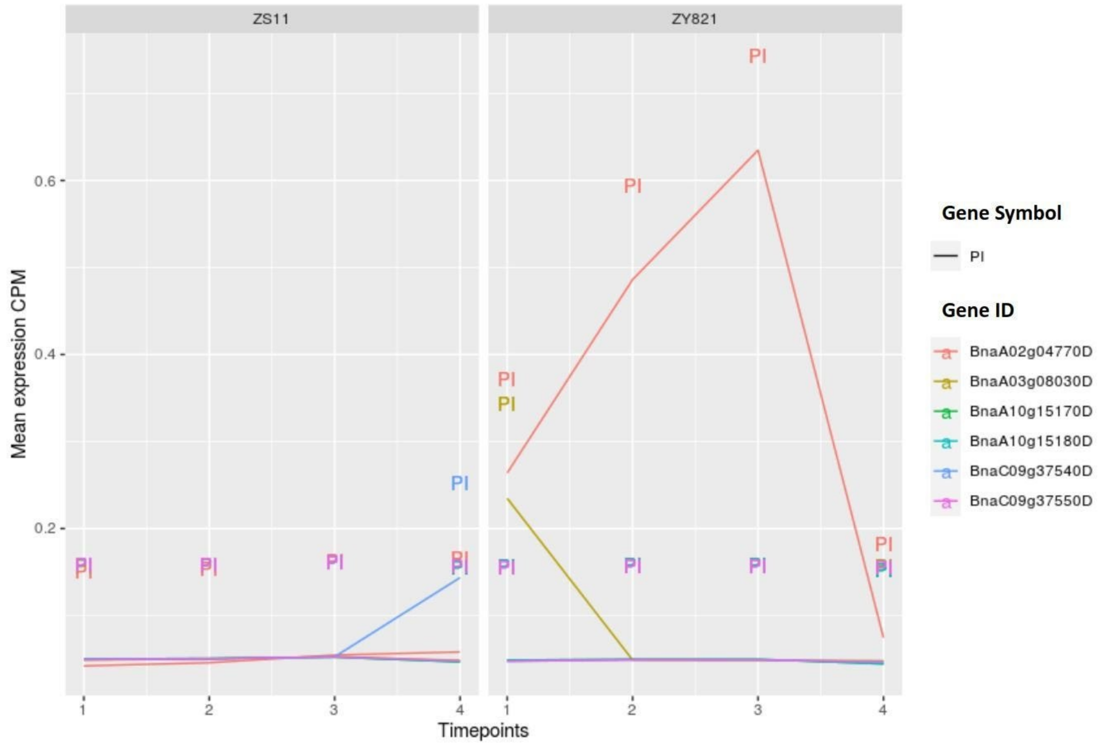

Figure S32

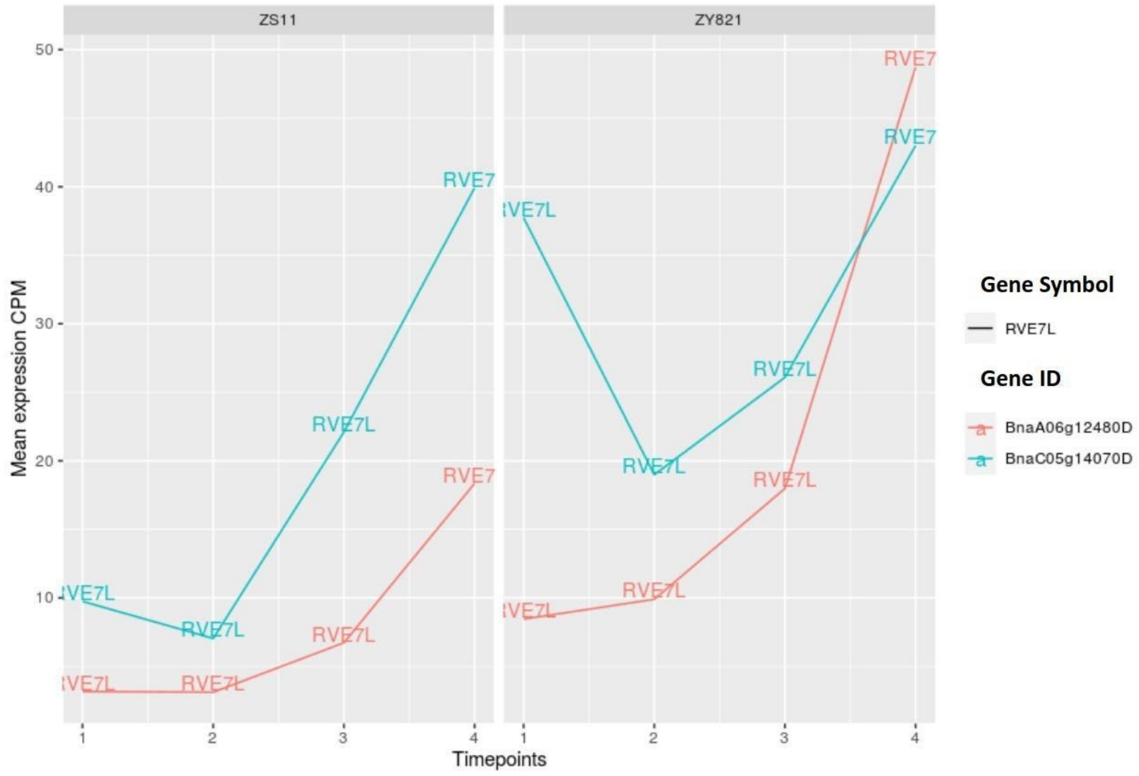

Figure S33

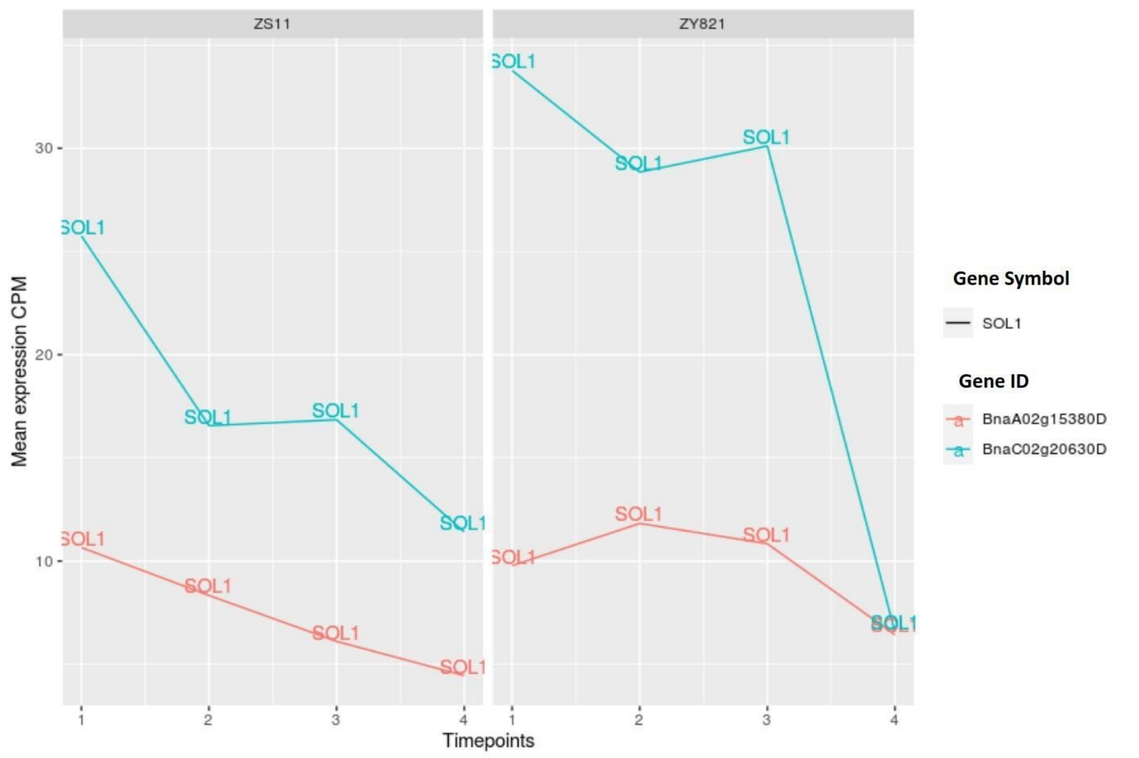

Figure S34

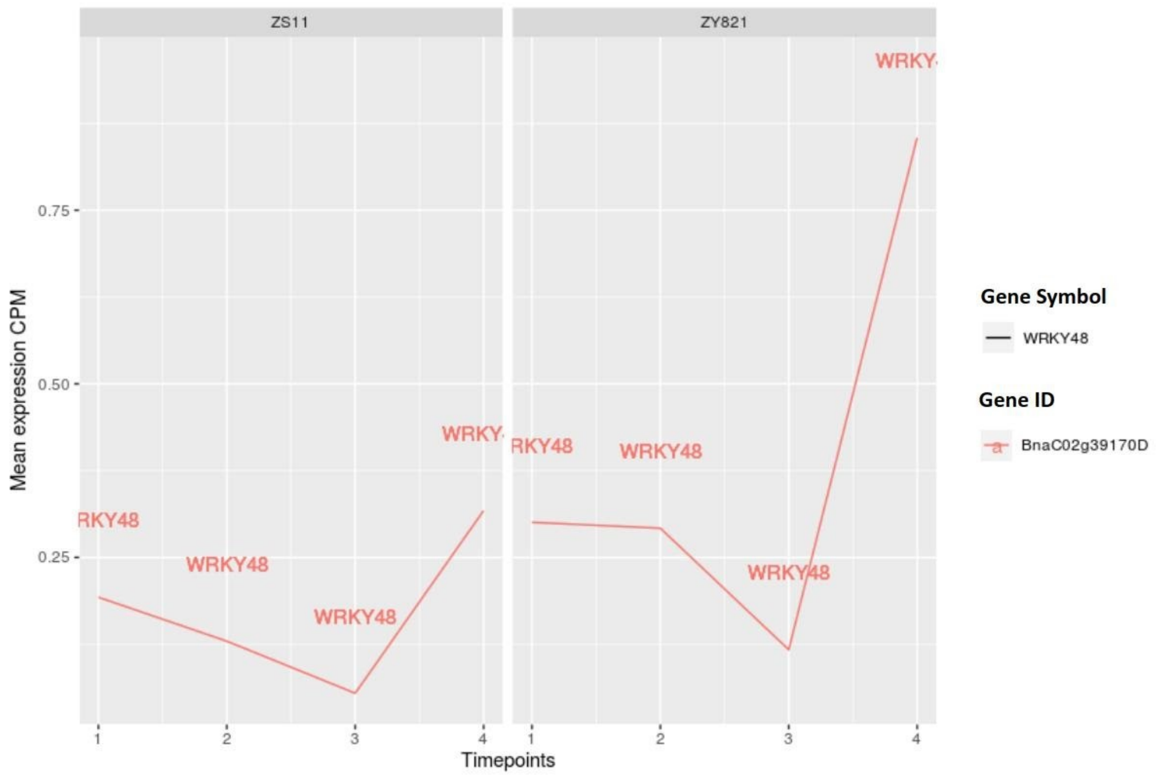

Figure S35

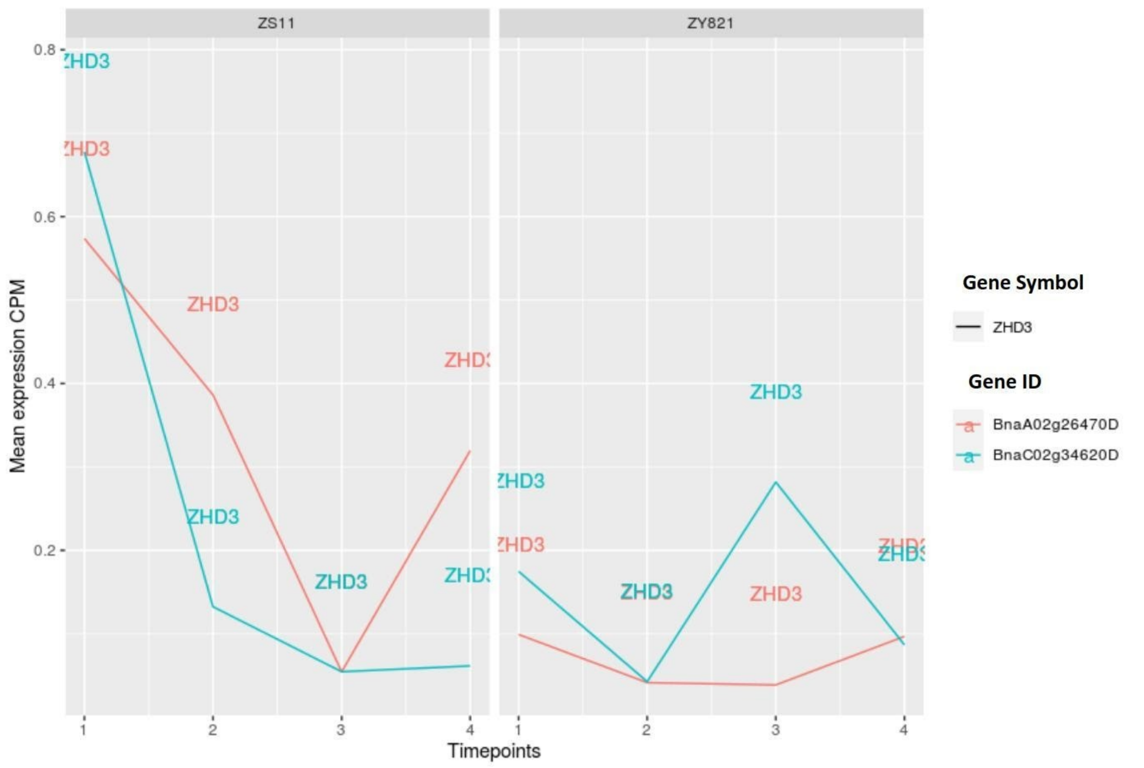

Figure S36

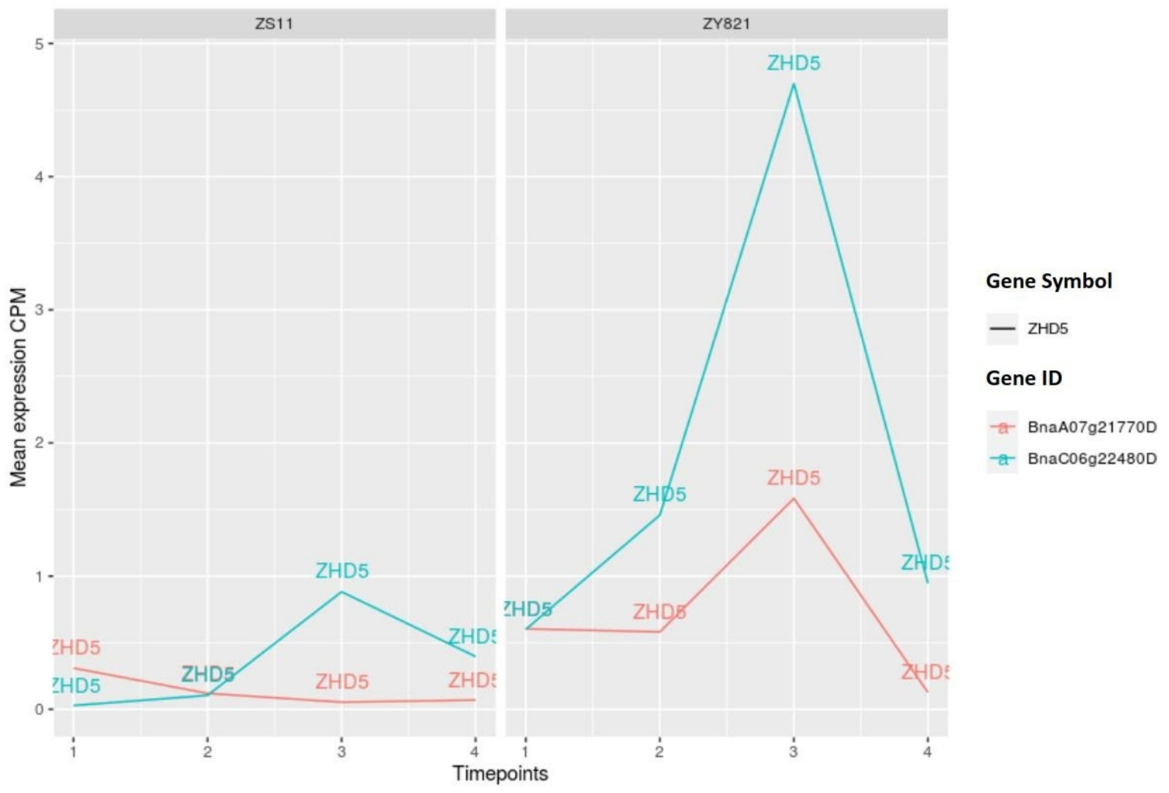

Supplement: Supplementary file 1 [file ijms-22-01033-s001.zip › SupplemetaryInformation.pdf]
