# Supplementary figures and images for: Unravelling the Complex Interplay of Transcription Factors Orchestrating Seed Oil Content in Brassica napus L
Source: Int J Mol Sci. 2021 Jan 21;22(3):1033. doi: 10.3390/ijms22031033 (PMC7864344; doi:10.3390/ijms22031033)

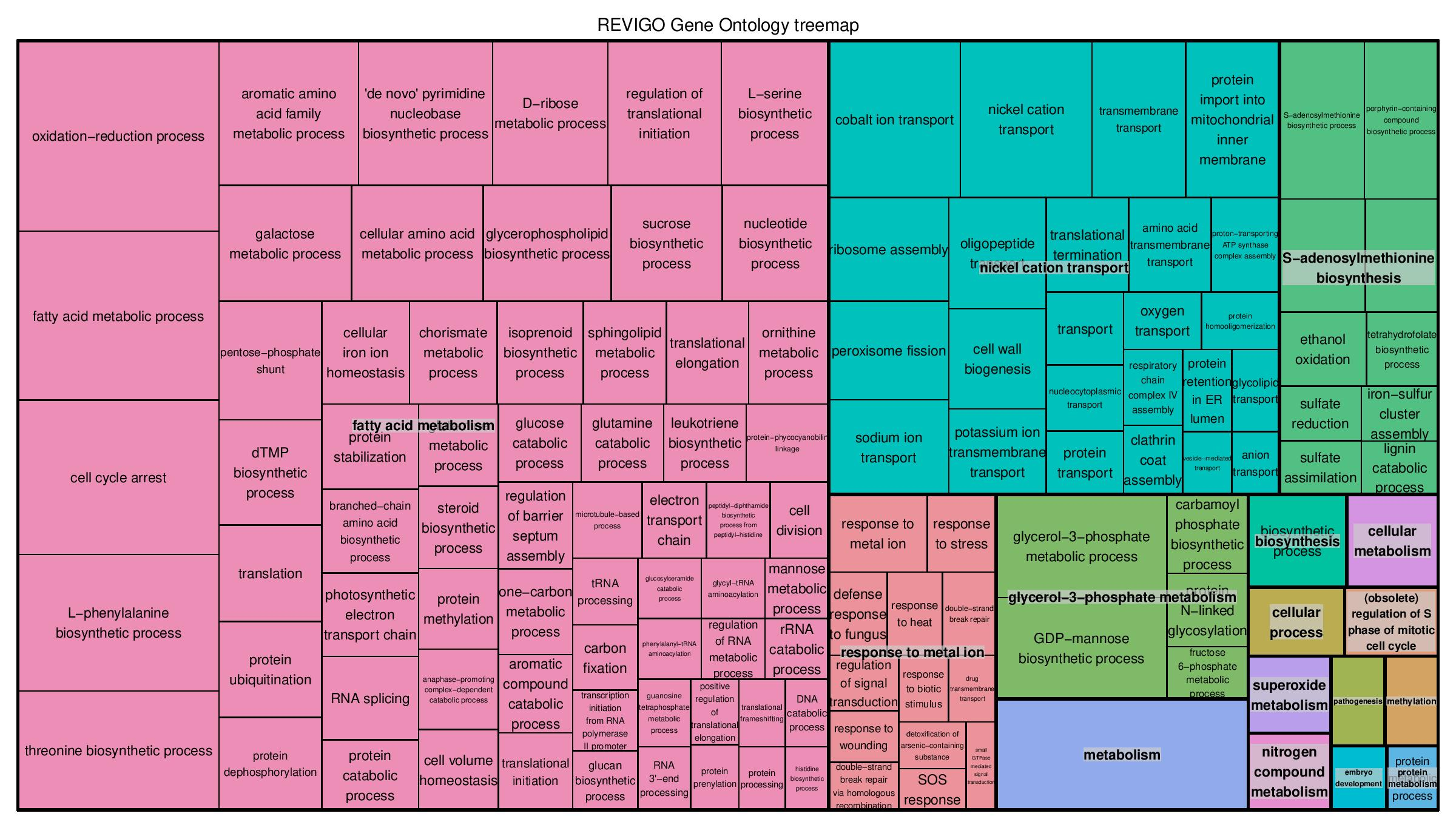

Supplement: Supplementary file 1 [file ijms-22-01033-s001.zip › Figure S1_zs_11_inc_BP_revigo_treemap-page-001.png]

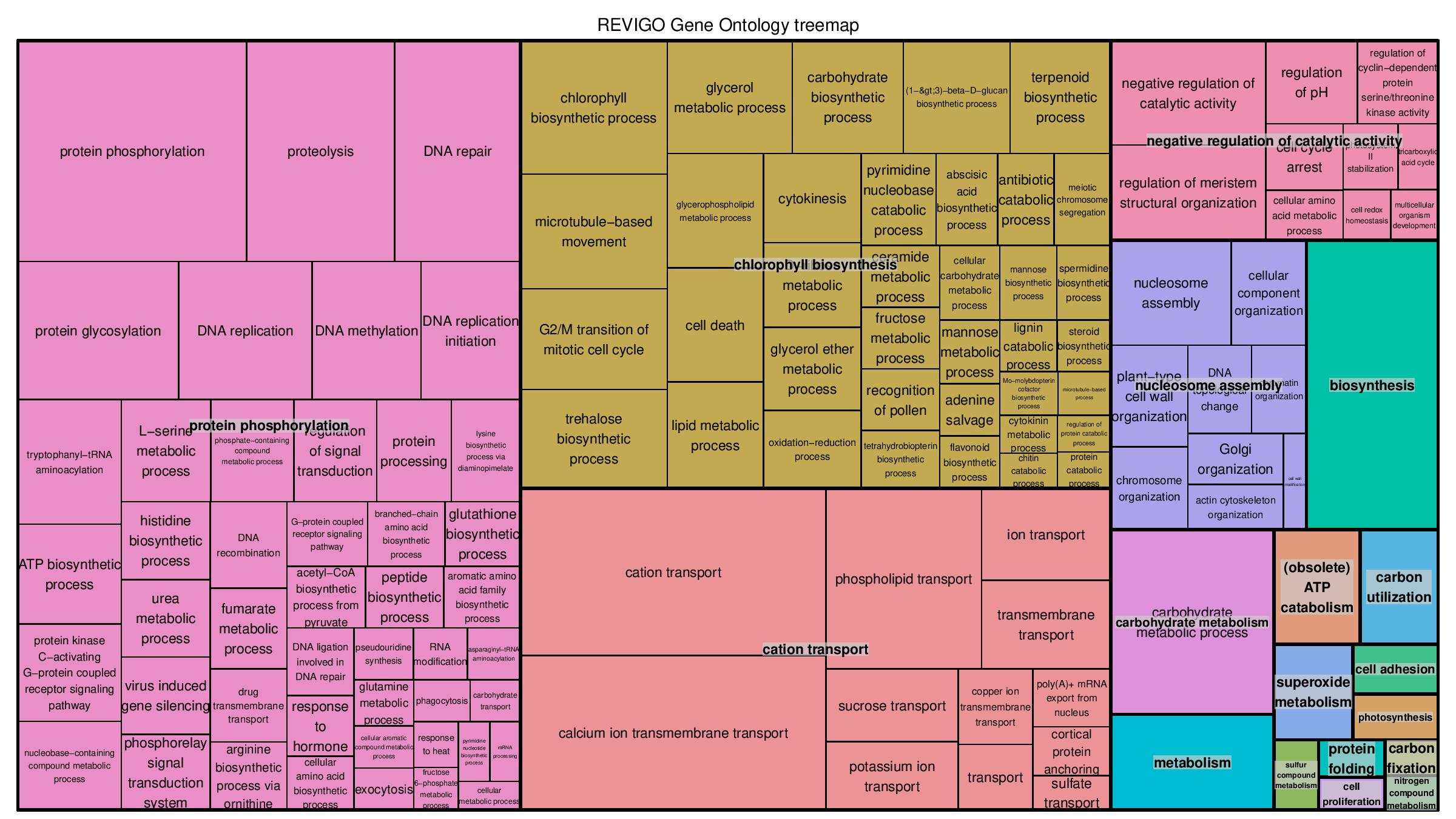

Supplement: Supplementary file 1 [file ijms-22-01033-s001.zip › Figure S2_zs_11_dec_BP_revigo_treemap-page-001.png]

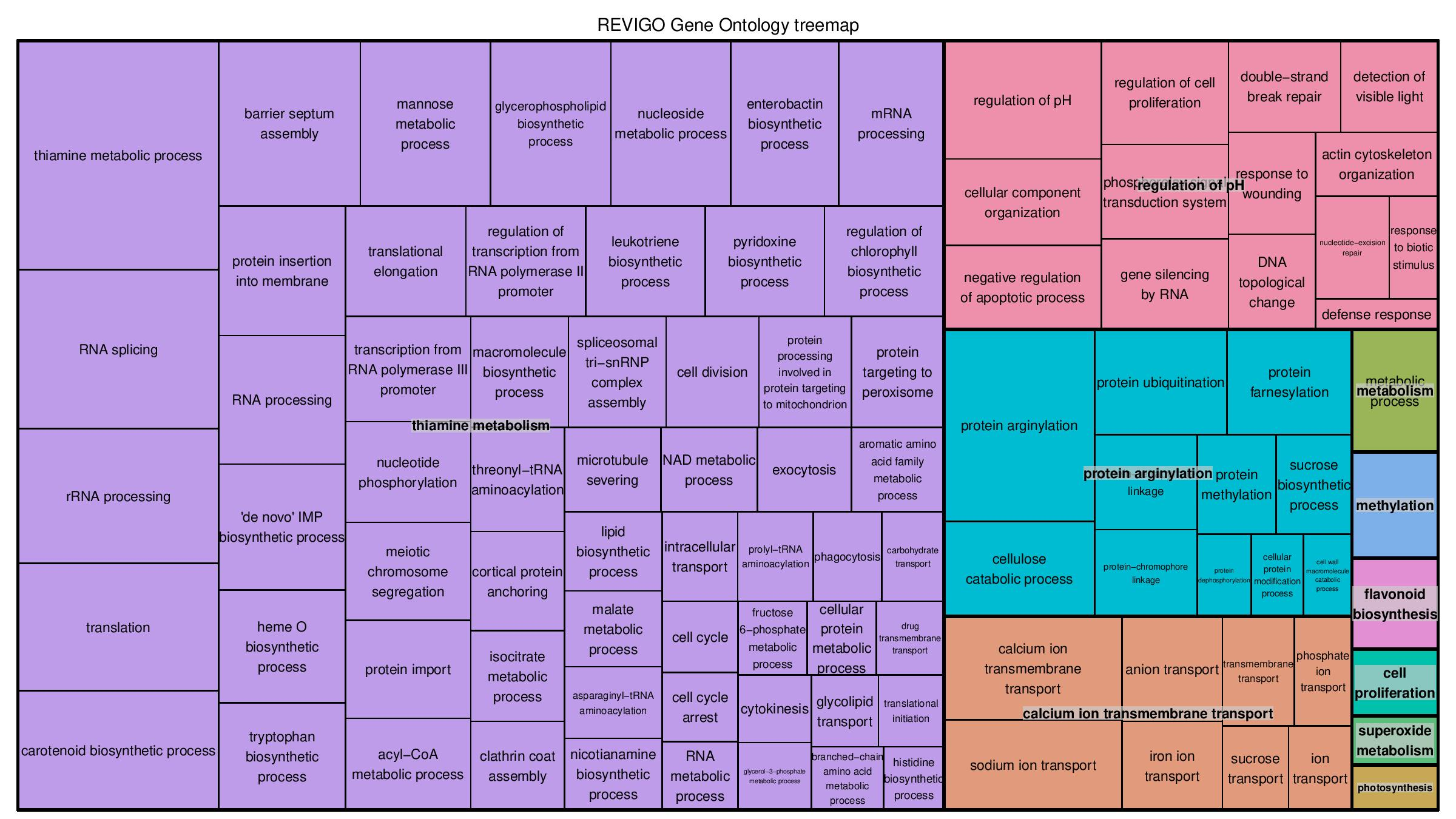

Supplement: Supplementary file 1 [file ijms-22-01033-s001.zip › Figure S3_ZY821_INC_BP_revigo_treemap-page-001.png]

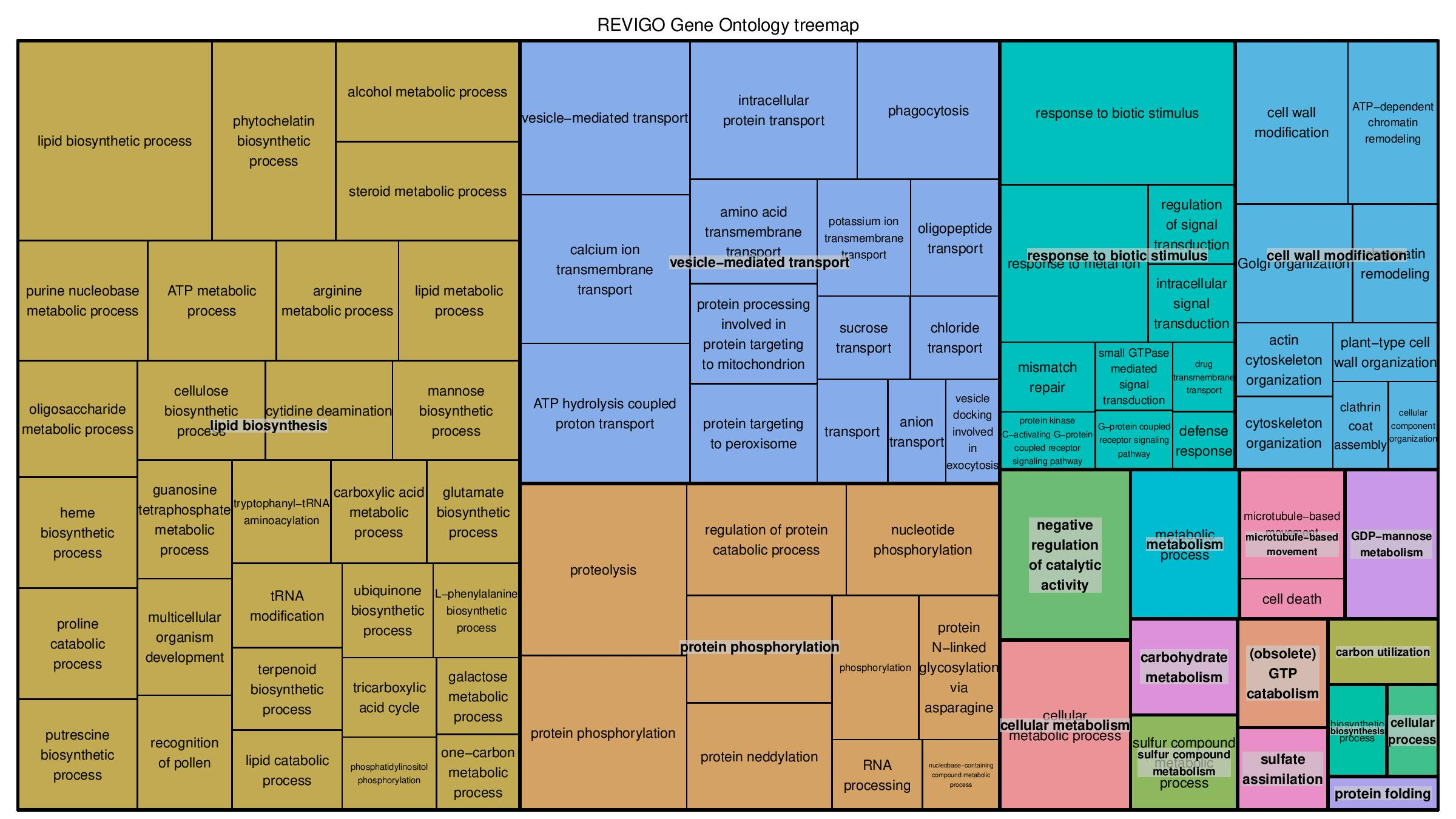

Supplement: Supplementary file 1 [file ijms-22-01033-s001.zip › Figure S4_ zy821_dec_BP_revigo_treemap-page-001.png]
